# GLP‑1 receptor agonist protects palmitate-induced insulin resistance in skeletal muscle cells by up-regulating sestrin2 to promote autophagy

**DOI:** 10.1038/s41598-023-36602-6

**Published:** 2023-06-09

**Authors:** Xue Tian, Yu Gao, Mowei Kong, Lihua Zhao, Enhong Xing, Qitian Sun, Jianqiu He, Yanan Lu, Zengbin Feng

**Affiliations:** 1 Department of Endocrinology, Affiliated Hospital of Chengde Medical University, Chengde, China; 2 Central Laboratory, Affiliated Hospital of Chengde Medical University, Chengde, China

**Keywords:** Cell biology, Drug discovery, Molecular biology

## Abstract

In this study, we aimed to determine whether liraglutide could effectively reduce insulin resistance (IR) by regulating Sestrin2 (SESN2) expression in L6 rat skeletal muscle cells by examining its interactions with SESN2, autophagy, and IR. L6 cells were incubated with liraglutide (10–1000 nM) in the presence of palmitate (PA; 0.6 mM), and cell viability was detected using the cell counting kit-8 (CCK-8) assay. IR-related and autophagy-related proteins were detected using western blotting, and IR and autophagy-related genes were analyzed using quantitative real-time polymerase chain reaction. Silencing SESN2 was used to inhibit the activities of SESN2. A reduction in insulin-stimulated glucose uptake was observed in PA-treated L6 cells, confirming IR. Meanwhile, PA decreased the levels of GLUT4 and phosphorylation of Akt and affected SESN2 expression. Further investigation revealed that autophagic activity decreased following PA treatment, but that liraglutide reversed this PA-induced reduction in autophagic activity. Additionally, silencing SESN2 inhibited the ability of liraglutide to up-regulate the expression of IR-related proteins and activate autophagy signals. In summary, the data showed that liraglutide improved PA-induced IR in L6 myotubes by increasing autophagy mediated by SESN2.

## Introduction

Many metabolic disorders, including fatty liver disease, obesity, and type 2 diabetes mellitus (T2DM), are associated with insulin resistance (IR). High plasma free fatty acid (FFA) levels have been identified as a risk factor for IR^1^. Excessive amounts of FFA in the blood results in the accumulation of lipid metabolites in the liver and muscles, worsening IR. As a result of many factors, including mitochondrial dysfunction, endoplasmic reticulum stress (ERS), and inflammation, FFA may cause IR in target tissues^2^. However, the molecular mechanisms of IR remain unclear. Increasing evidence suggests that autophagy plays a role in IR development.

Autophagy is a catabolic process that is important to maintain homeostasis and respond to cell stress^3^. Increased autophagy in the target tissues of insulin improves insulin sensitivity by decreasing ERS^4^. Suppression of autophagy causes IR in mouse liver tissues. Insulin activity in liver tissues increases when autophagy is restored in obese mice^5^. In addition, Cheng et al.^6^ found that autophagy in skeletal muscle of high-fat-fed (HFD) mice decreased. Exercise can promote autophagy by activating adenosine monophosphate activated protein kinase (AMPK) / peroxisome proliferator-activated receptor γ coactivator-1a-1a (PGC-1a) pathway, thus improving the insulin sensitivity of skeletal muscle damage in HFD mice. These findings suggest that autophagy plays a role in IR, and its regulation is a valuable strategy to treat and prevent T2DM.

Sestrin2 (SESN2) belongs to the Sestrin family of proteins and fights numerous physiological stresses, including DNA damage, hypoxia, and oxidative stress^7^. Recent reports have shown that SESN2 activates autophagy by inhibiting the mammalian target rapamycin complex 1 (mTORC1) through both AMPK-dependent and AMPK-independent mechanisms, which play key roles in eliminating damaged organelles from stressed cells^8^. Recently, a study reported that exercise increases insulin sensitivity by interacting with AMPK and SESN2^9^. Moreover, SESN2-/- mice display impaired insulin responsiveness and impaired insulin-induced PI3K-Akt signaling in the liver^10^. Therefore, regulating SESN2 activity could be an alternative method to prevent IR, obesity, and diabetes.

Liraglutide, an analog of glucagon-like peptide-1 (GLP-1) derived from human GLP-1, has a sustained effect on lowering blood glucose and lipid levels. Liraglutide has been found to alleviate IR by inhibiting insulin receptor substrate 1 (IRS1) serine phosphorylation in skeletal muscle cells treated with palmitate (PA)^11^. Liraglutide delays INS-1 cell apoptosis induced by FFA by activating autophagy^12^. Similarly, Jing et al. demonstrated that liraglutide-induced autophagy protects INS-1 cells from FFA-induced apoptosis, improving the survival of INS-1 cells^13^. Additionally, liraglutide ameliorated obesity-related non-alcoholic fatty liver disease (NAFLD) by activating SESN2 in HFD mice^14^.

However, no evidence indicates that SESN2-mediated autophagy activation is associated with the protective effects of liraglutide on PA-induced IR in skeletal muscle cells. The aim of this study was to determine whether liraglutide improves IR by increasing SESN2 expression in skeletal muscle cells, thus offering new clinical options to treat T2DM.

## Materials and methods

### Cells and culture

The rat L6 myoblast line was donated by the Clinical Medical Research Center of Hebei Provincial People’s Hospital. The cells were cultured in high glucose Dulbecco’s modified Eagle’s medium (DMEM) (Thermo Fisher, USA) supplemented with 10% fetal bovine serum (FBS; BI, Israel), penicillin 100 IU/mL, streptomycin 100 μg/mL at 37 °C with 5% CO_2_. When L6 cells reached 70%–80% confluence, DMEM containing 2% FBS was used to induce L6 cells to differentiate into myotubes for 5–6 days.

### Palmitate solution preparation

Palmitate solution was prepared as previously described^15^. A magnetic stirrer was used to mix palmitate in 100% ethanol at 70 °C, then dilute it in DMEM with bovine serum albumin at 40 °C with 2% fatty acid-free bovine serum albumin (BSA). PA was treated for 16 h with concentrations of 0.2 mM, 0.4 mM, 0.6 mM, and 0.8 mM on differentiated myotubes, followed by exposure to 100 nM insulin for 30 min. In the control treatment, 2% fatty-acid-free BSA-DMEM solutions were prepared by adding equal amounts of ethanol. For autophagy inhibition experiments, 3-MA (5 mM), an autophagy inhibitor, was added 1 h prior to PA treatment.

### Cell viability

The viability of L6 cells was measured using the cell counting kit-8 (CCK-8) assay (Meilunbio, Dalian, China). L6 cells were seeded in 96-well plates at a density of 6 × 10^3^ cells/well and cultured for differentiation. After myotube formation, the cells were cultured with different concentrations of liraglutide and PA for 16 h. Five duplicate wells and blank controls were used in the experiment. After the supernatant was removed, the culture medium containing 10%CCK-8 reagent was added and incubated at 37 ℃ for 3 h. When the culture medium color changed to dark orange, the optical density was measured at 450 nm (OD450).

### Glucose content measurement

The glucose content was determined by the GOD-POD (glucose oxidase–peroxidase) (Applygen Technologies, Beijing, China) micromethod according to the instructions. L6 cells were cultured in 96-well plates (5 × 10^4^/well) at 37 °C in an incubator with 5% CO_2_, and each group was provided with five replicate wells. The supernatants were collected after cell treatment; 5 μL of the supernatant was taken for testing and the optical absorbance measured at 505 nm. Supernatant glucose concentration was determined as directed by the manufacturer.

### Cell transfection

Small interfering RNA (siRNA) oligonucleotides specific for SESN2 were designed by zsgentech (Tianjin, China). In order to suppress gene expression, we transfected cells using Lipofectamine 3000 (Invitrogen, USA) as directed by the manufacturer. SESN2-siRNA or non-silencing siRNA was diluted in transfection medium to a final concentration of 70 nM, mixed with siRNA transfection reagent, incubated for 20 min at room temperature, and added to the cells. Transfection solution was incubated with L6 myotubes for 10 h, and then the medium was replaced with fresh medium. After 24 h of culture, the cells were exposed to the specified treatment.

### Western blotting analysis

L6 myotubes were lysed, sonicated, and homogenized in radioimmunoprecipitation assay (RIPA) lysis buffer, supplemented with protease and phosphatase inhibitors. Determination of protein concentration in supernatant using BCA protein Assay Kit (Applygen Technologies, Beijing, China), and equal amounts of total protein underwent 10% SDS-PAGE and were transferred to polyvinylidene difluoride membranes, which were blocked with 5% non-fat milk, and incubated with different primary antibodies overnight at 4 °C. The anti-P62, anti-GLUT4, anti-LC3 II/LC3 I, anti-pAkt and anti-SESN2 antibodies were purchased from Affinity Biosciences (USA). The anti-Akt antibody was obtained from HuaBio (Hangzhou, China). After incubation with secondary antibodies(Seracare, USA) conjugated to horseradish peroxidase, membranes were visualized using an enhanced chemiluminescence system. The ImageJ software was used for densitometric analysis.

### Quantitative reverse transcriptase-polymerase chain reaction (RT-qPCR)

Total RNA was isolated with Superbrilliant™ 6 min High-quality RNA Extraction Kit (zsgentech, Tianjin, China), according to the manufacturer's instructions. cDNA synthesis was performed using the Supersmart™ 6 min 1st Strand cDNA Synthesis kit (Zsgentech, Tianjin, China) according to the manufacturer’s instructions using 1 µg of RNA as a template. SupersmartTM2 × ZAPA3G SYBR Green qPCR Mix (Zsgentech, Tianjin, China) and cobas® z 480 Automatic Fluorescence Quantitative PCR analyzer (Roche, Germany) were used for RT-qPCR, and the reaction conditions were set as follows: at 95 ℃ for 5 min, 40 cycles at 95 ℃ for 10 s, at 60℃ for 20 s, and at 70 ℃ for 10 s. GAPDH served as an internal control. The 2^−ΔΔCT^ method was used to calculate the relative expression. All primers used for RT-qPCR are listed in Table 1.

**Table 1:** Primer sequences used for quantitative real-time PCR.

Gene	Forward	Reverse
LC3II	5′-CTGCTTCCTGCTACCTGCAT-3′	5′-GGGACATGACGACGTACACA-3′
SESN2	5′-AGCTGGAGAAGACGGAAAGC-3′	5′-CCAAACGTGGGGTCCTCTAC-3′
P62	5′-GCACCCCAACGTGATTTGTG-3′	5′-GTGCCCATGTTTCAGCTTCC-3′
GLUT4	5′-GATGCCGTCGGGTTTCCAGCA-3′	5′-TGAGGGTGCCTTGTGGGATGG-3′
GAPDH	5′-GGCACAGTCAAGGCTTGAAGAG-3′	5′-ATGGTTGGTGAAGAGAGCGCCAGTA-3′

### Statistical analysis

SPSS 26.0 and GraphPad Prism 8.0 software were used for statistical analyses. Data are presented as the mean ± standard deviation(SD) for independent experiments. Multiple group differences were assessed by one-way analysis of variance (ANOVA) followed by the least significant difference (LSD) post-hoc test. Statistical significance was set at P < 0.05.

## Results

### Liraglutide protects against L6 myotube death induced by PA

**Figure 1:**
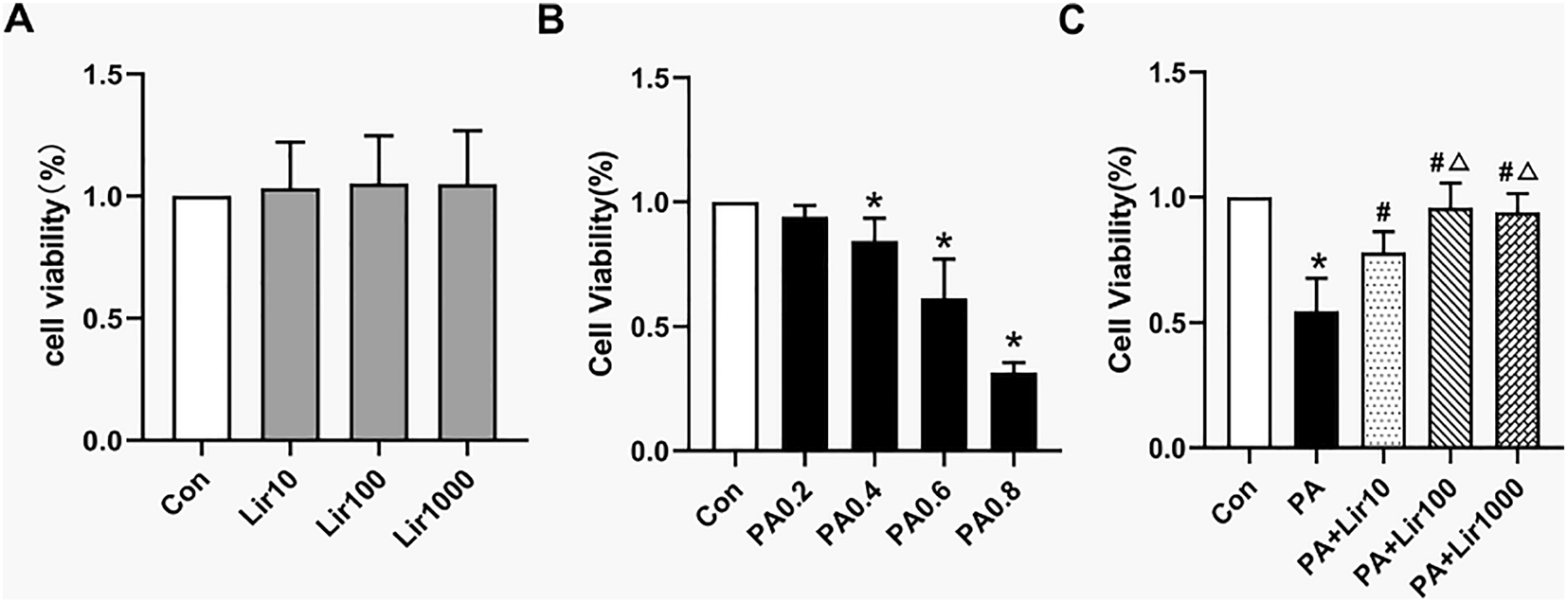
Effects of liraglutide on palmitate (PA)-induced cell viability in L6 myotubes. (**A**) Cell viability was analyzed by cell counting kit 8 (CCK-8) assay. The cells were treated with various concentrations of liraglutide (10, 100, 1000 nM) for 16 h. (**B**) Viability of L6 myotubes in response to different doses of PA (0.2, 0.4, 0.6, 0.8 mM) for 16 h measured by CCK-8 assay. (**C**) Cells were incubated with 0.6 mM PA for 16 h with or without liraglutide at different concentrations. *P < 0.05 vs control; #P < 0.05 vs PA; △P < 0.05 vs PA + Lir10.

The results of the CCK-8 assay showed that there was no obvious decline in cell viability between liraglutide and control groups(Fig. 1A). L6 myotubes were treated with various concentrations of PA in order to determine the effect of PA on their survival rate. After 16 h of treatment, PA did not affect the survival of L6 myotubes when concentrations were ≤ 0.2 mM; however, their survival was significantly suppressed when PA concentrations were ≥ 0.4 mM (Fig. 1B). Therefore, 0.6 mM PA concentration was selected for subsequent experiments. Taking into account these results and previous research^16,17^, The protective effects of liraglutide against PA-induced lipotoxicity were evaluated at doses of 10, 100, and 1000 nM. There was a significant decrease in the death of L6 myotubes induced by PA following liraglutide treatment in a dose-dependent manner (Fig. 1C). These results indicate that liraglutide exhibits strong protective properties against PA-induced skeletal muscle lipotoxicity.

### Liraglutide ameliorated PA-induced decrease in glucose uptake and IR in L6 myotubes

**Figure 2:**
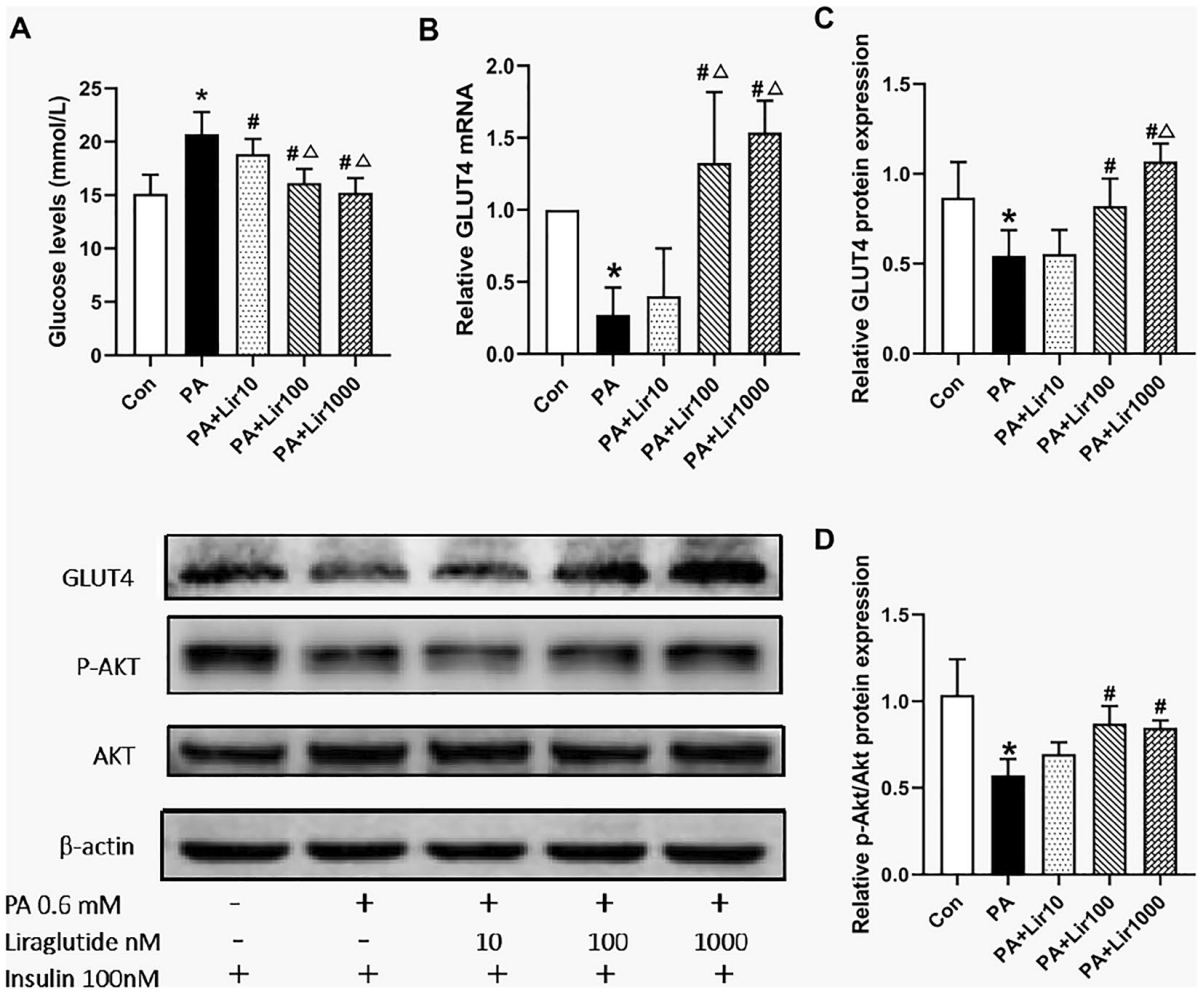
Liraglutide improves insulin resistance (IR) in PA-induced L6 myotubes. (**A**) The glucose oxidase–peroxidase micromethod was used to determine the glucose level in the cell supernatant. (B) The mRNA levels of GLUT4 in L6 myotubes were determined by quantitative reverse transcriptase-polymerase chain reaction. (**C**) and (**D**) The protein levels of GLUT4 and p-Akt/Akt in L6 myotubes were determined using western blotting. The results are expressed as means ± S.D. of three independent experiments. *P < 0.05 vs con; #P < 0.05 vs PA; △P < 0.05 vs Lir10.

Liraglutide was applied to PA-induced L6 myotubes at various concentrations to determine its effects on glucose uptake and IR. The PA-induced groups showed significantly higher levels of glucose in the supernatant compared to the control group (Fig. 2A). However, GLUT-4 mRNA expression was lower in L6 myotubes compared with the control group (Fig. 2B). After treatment with liraglutide, the glucose content in the culture medium significantly decreased compared with that in the IR cells. Also, GLUT-4 expression was significantly higher in the Lir100 and Lir1000 groups than in the IR cells. This finding was further analyzed using western blotting. As shown in Fig. 2C, compared to the control group levels, the levels of GLUT-4 and p-Akt/Akt were significantly down-regulated after PA treatment. However, liraglutide reversed the decrease in GLUT-4 protein expression and p-Akt/Akt ratio induced by PA in L6 myotubes in a dose-dependent manner (Fig. 2D).

### Effects of liraglutide on PA-induced autophagy and SESN2 expression in L6 cells

**Figure 3:**
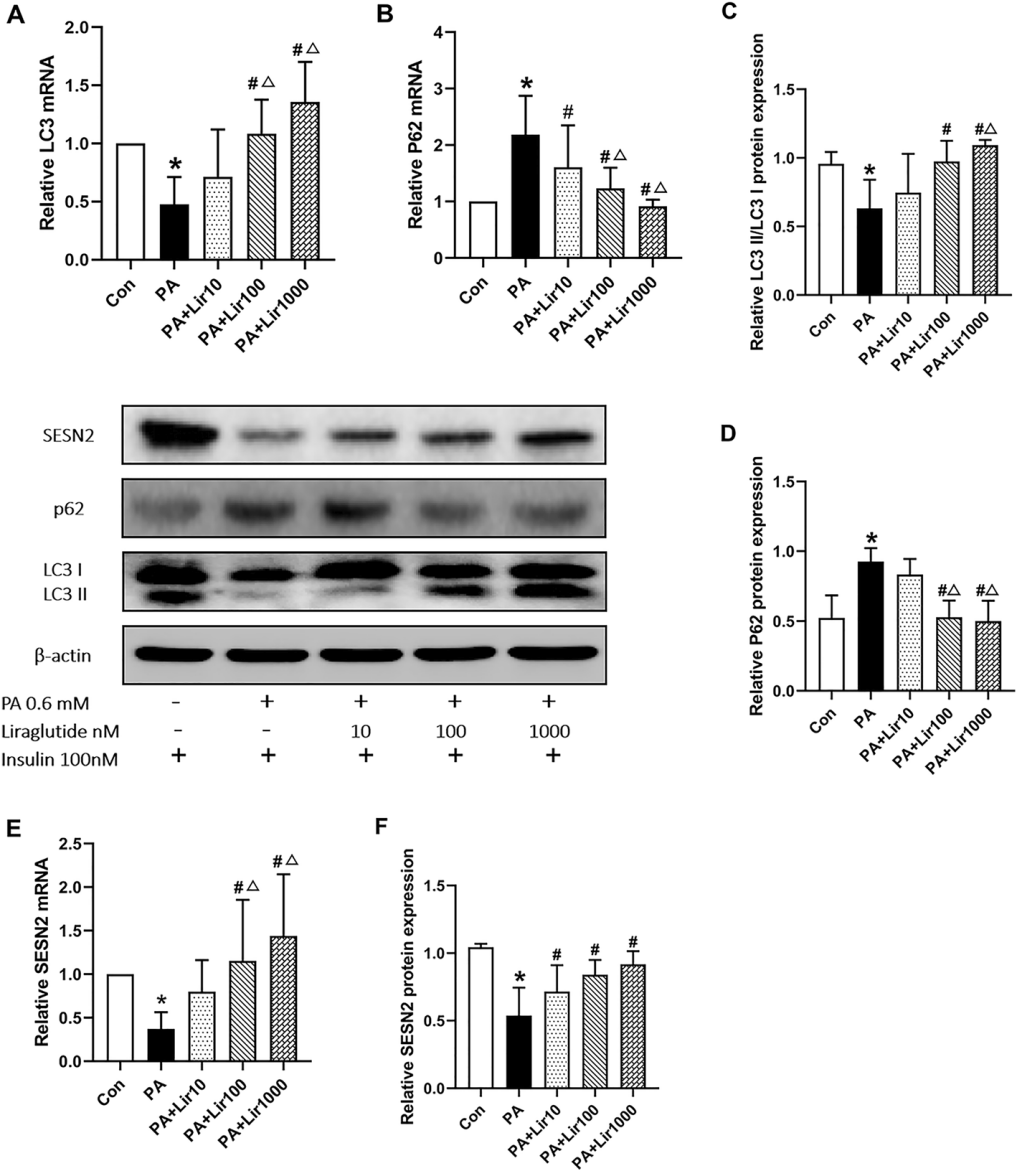
Liraglutide activates PA-induced L6 myotubes autophagy and up-regulates SESN2 expression. (**A**) and (**B**) The mRNA levels of LC3II and P62 in L6 myotubes were determined by RT-qPCR. (**C**) and (**D**) The protein levels of LC3II/LC3I and P62 in L6 myotubes were determined using western blotting. The (**E**) mRNA and (**F**) protein levels of SESN2 in L6 myotubes were determined using RT-qPCR and western blotting. The results are expressed as means ± S.D. of three independent experiments. *P < 0.05 vs con; #P < 0.05 vs PA; △P < 0.05 vs Lir10.

We found that LC3II expression decreased in the PA group compared to the control group (Fig. 3A,C), while P62 expression increased significantly in the PA group compared to the control group (Fig. 3B,D). After intervention with liraglutide, the expression of LC3II significantly increased, and that of P62 significantly decreased relative to that in the PA group. These results demonstrated that liraglutide inhibited PA-induced IR in L6 myotubes, which may be attributed to its ability to enhance autophagy. In addition, the mRNA and protein levels of SESN2 in the PA group were lower than those in the control group, and liraglutide treatment alleviated this decrease in SESN2 expression caused by PA in a dose-dependent manner (Fig. 3E,F).

Liraglutide increased the expression levels of GLUT-4 and p-Akt and activated autophagy in a dose-dependent manner. The Lir100 and Lir1000 groups showed more significant changes than the Lir10 group (P < 0.05), whereas there were no statistically significant differences between the Lir1000 and Lir100 groups. Therefore, a liraglutide concentration of 100 nM was selected as the therapeutic concentration in subsequent studies.

**Figure 4:**
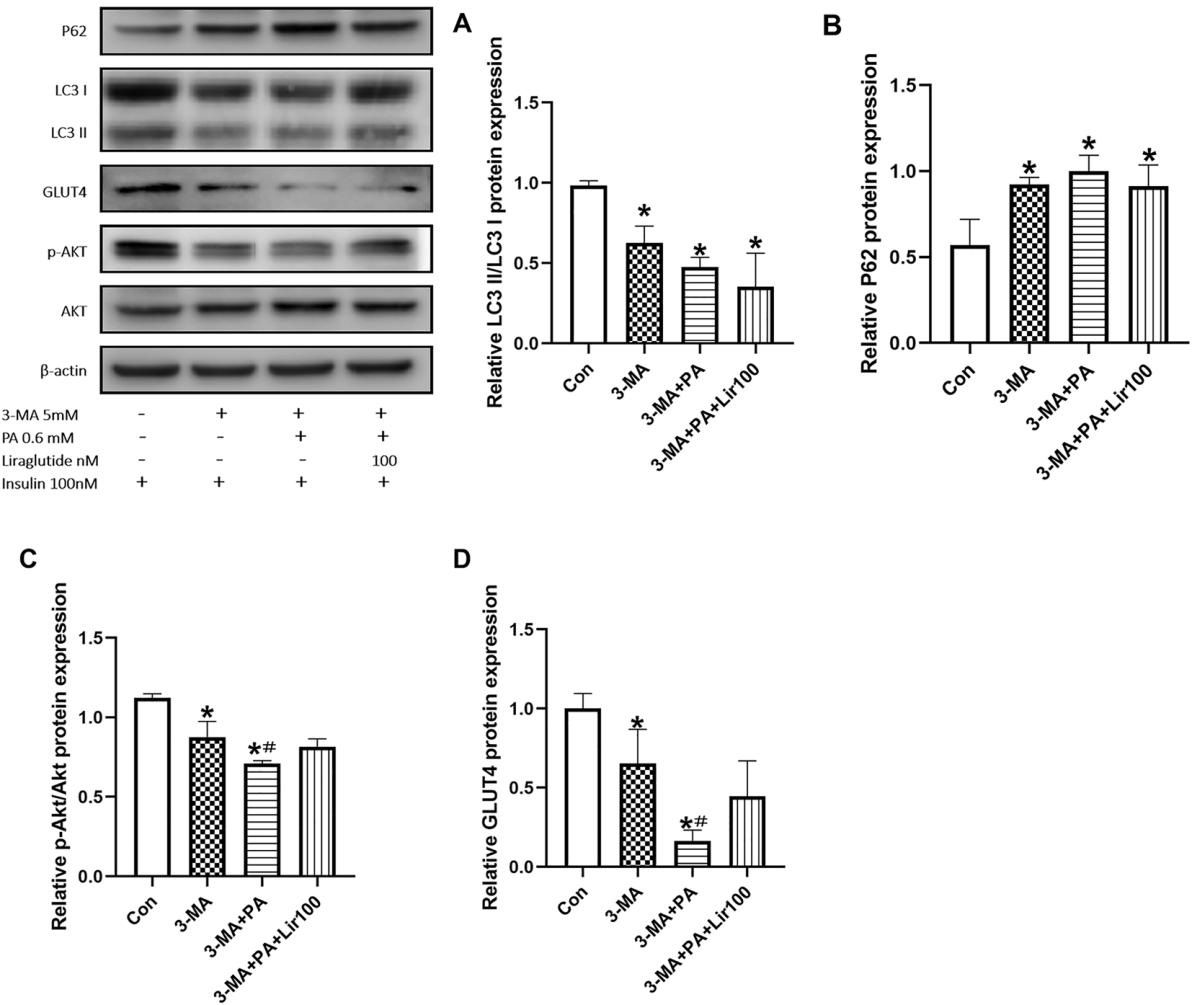
Liraglutide attenuated IR by inducing autophagy activation in myotubes. The protein levels of LC3II/LC3I (**A**), P62 (**B**), p-Akt/Akt (**C**) and GLUT4 (**D**), in L6 myotubes were determined using western blotting. The results are expressed as means ± S.D. of three independent experiments. *P < 0.05 vs CON, #p < 0.05 vs 3-MA treatment alone.

The autophagy inhibitor 3-MA was used to investigate whether liralutide improves IR by improving autophagy. Upon treatment with 3-MA, LC3II/LC3I expression was down-regulated and p62 expression increased, indicating an inhibition of autophagy(Fig. 4A,B). As shown in Fig. 4C,D, IR myotubes with suppressed autophagy showed p-Akt/Akt and GLUT-4 levels significantly decreased. Correspondingly, under the condition of autophagy inhibition, the increase of p-Akt/Akt and GLUT-4 induced by liralutide was significantly reduced.

### Liraglutide improved the autophagy induced by PA via SESN2

**Figure 5:**
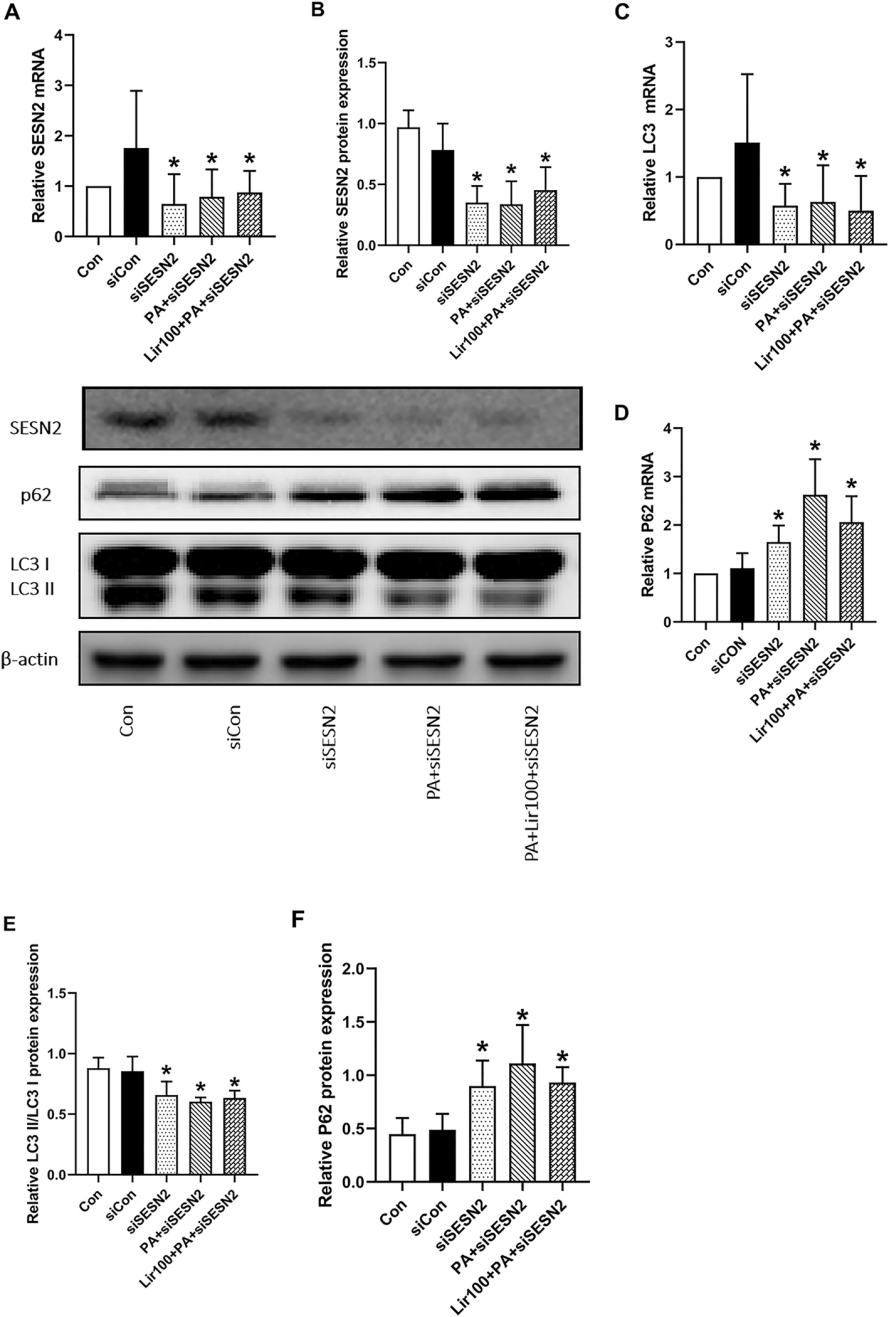
Lilarutide ameliorates palmitate PA-induced IR and autophagy via SESN2. The (**A**) mRNA and (**B**) protein levels of SESN2 in L6 myotubes were determined by quantitative reverse transcriptase-polymerase chain reaction (RT-qPCR) and western blot. (**C**) and (**D**) The protein levels of LC3B and P62 in L6 myotubes were determined using RT-qPCR. (**E**) and (**F**) The mRNA levels of LC3II/LC3I and P62 in L6 myotubes were determined using western blotting. The results are expressed as means ± S.D. of three independent experiments. *P < 0.05 vs siCON.

We knocked down SESN2 expression in L6 myotubes in order to confirm that SESN2 is involved in autophagy. L6 myotubes transfected with siRNA targeting SESN2 were found to be successfully suppressed in terms of SESN2 expression (Fig. 5A,B). The mRNA expression of LC3II was decreased and the expression of P62 was increased in the siSESN2 group and the siSESN2 + PA group compared with the siCON group. However, PCR showed that the inhibitive effects of liraglutide against PA-induced decreases in SESN2, LC3II and increases in P62 expression were abrogated in L6 cells transfected with SESN2 siRNA (Fig. 5C,D). The protein expression of LC3II/LC3I and P62 altered in a similar manner (Fig. 5E,F). There were no significant difference in the expression of SESN2, LC3II and p62 among siSESN2, siSESN2 + PA and siSESN2 + PA + liraglutide groups. The proteins associated with autophagy were downregulated in L6 myotubes after SESN2 was silenced. Importantly, liraglutide treatment did not stimulate autophagy in SESN2-silenced L6 myotubes, thus suggesting that SESN2 plays a role in autophagy stimulation by liraglutide.

### SESN2-regulated autophagy is mechanistically involved in liraglutide-mediated alleviation of IR

**Figure 6:**
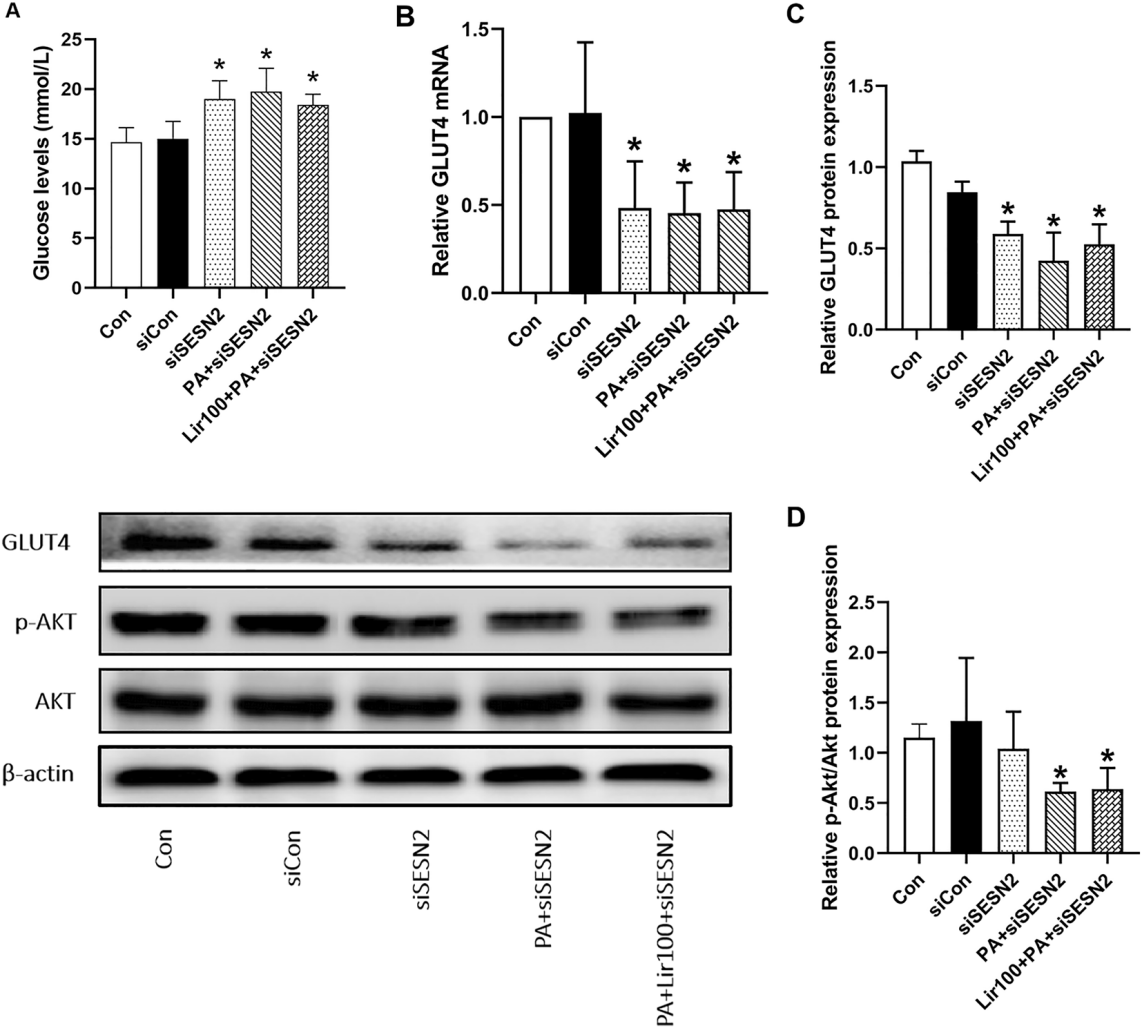
Liraglutide regulated L6 myotube IR by activating SESN2. (**A**) The glucose oxidase–peroxidase micromethod was used to determine the level of glucose in the cell supernatant. (**B**) The mRNA levels of GLUT4 in L6 myotubes were determined using quantitative reverse transcriptase-polymerase chain reaction. (**C**) and (**D**) The protein levels of GLUT4 and p-Akt/Akt in L6 myotubes were determined using western blotting. The results are expressed as means ± S.D. of three independent experiments. *P < 0.05 vs siCON.

As part of our current study, we examined whether SESN2-mediated autophagy may contribute to improve IR by liraglutide. Compared with the levels of siCON group, the levels of glucose in the cell supernatant (Fig. 6A) in L6 myotubes were significantly increased, and the mRNA and protein expression of GLUT-4 were significantly decreased (Fig. 6B,C) in the treated groups (siSESN2, PA + siSESN2, and Lir100 + PA + siSESN2) , whereas the protein expression ratio of p-Akt/Akt decrease in the siSESN2 group , the difference was not statistically significan; but significantly decreased in the PA + siSESN2 and Lir100 + PA + siSESN2 groups (Fig. 6D). Furthermore, there were no significant difference among siSESN2, siSESN2 + PA and siSESN2 + PA + lir100 groups. The results showed that the inhibitory effects of liraglutide against PA-induced reductions in Akt phosphorylation and decreases in the protein and mRNA expression of GLUT4 were abrogated in L6 cells transfected with SESN2 siRNA. Collectively, SESN2 is required for liraglutide improvement skeletal muscle IR in PA-exposed L6 cells.

## Discussion

IR is closely associated with several diseases such as dyslipidemia, hyperglycemia, hypertension, inflammation, and endothelial dysfunction^18^. As the body’s primary system for absorbing and removing glucose, the skeletal muscle consumes ~ 80% of the glucose produced by the body. Therefore, insulin resistance and type 2 diabetes are characterized by a reduction in insulin-stimulated glucose uptake in the skeletal muscles^19^. In the current study, we used PA to mimic the in vivo high-fat environment and induce IR in L6 myotubes, and investigated the effect of liraglutide on the IR of myotubes.

In the skeletal muscle, GLUT4 is primarily found in intracellular storage vesicles and is essential for maintaining glucose metabolism homeostasis as well as insulin sensitivity. Akt is activated as a result of insulin or insulin growth factor signaling and plays a vital role in GLUT4 translocation^20^. Activation of Akt in the skeletal muscle has been shown to promote glucose uptake by increasing GLUT4 translocation and expression^21^. We successfully established a PA-induced IR model in L6 myotubes, characterized by a significant decrease in intracellular glucose uptake. GLUT4 expression and Akt phosphorylation were significantly down-regulated by PA treatment.

Liraglutide, a GLP-1 analog,plays a well-defined role in the pancreas, where it enhances glucose-stimulated insulin secretion^22^. In addition, there is evidence that GLP-1 has effects on adipose tissue and skeletal muscle that are extra-pancreatic^23,24^. When transgenic mice overexpress muscle-specific GLP-1 during a high-fat diet challenge, weight gain is reduced and lipid profiles are improved^25^. GLP-1 up-regulates the translocation and expression of GLUT4 in rats with spontaneously hypertensive hearts and skeletal muscles^26^. Additionally, liraglutide increased GLUT4 expression in the liver and increased GLUT4 expression in liver tissue and skeletal muscle in diabetic KKAy mice^27^. Based on the findings of our study, GLP-1 restores the decreases in glucose uptake and the expression of GLUT4 and p-Akt caused by PA, which is consistent with previous reports^28^.

Autophagy is a highly conserved regulatory process responsible for helping cells recycle damaged organelles, facilitating the maintenance of homeostasis^29^. Lipid overload has been suggested to cause the loss or downregulation of autophagy, leading to fat accumulation, mitochondrial disorders, inflammation, and decreased pancreatic β-cell quality, all of which may eventually lead to IR and T2DM^30^. Asie et al.^31^ found that PA-induced defect of autophagic flux leads to elevated inflammatory cytokine expression in the skeletal muscle cells by regulating the oxidative stress process. And low grade inflammation was associated with obesity and insulin resistance. Researchers have demonstrated that enhanced autophagy can protect skeletal muscle from lipotoxicity, suggesting that autophagy may provide protection against T2DM^32^. Thus, LC3II/LC3I and p62 proteins associated with autophagy flux were evaluated. In L6 myotubes, we observed that PA decreased the expression of LC3II/LC3I and increased the expression of P62. Li and Wang^33,34^ reported that PA treatment decreases LC3II/LC3I expression and increases P62 expression in hepatocytes and C2C12 myotubes, which is in line with our study results. Furthermore, liraglutide protects islet β-cells from FFA by activating autophagy^12,13^. Our study showed that liraglutide increased autophagy in L6 myotubes treated with PA in a dose-dependent manner. Therefore, we speculated that liraglutide ameliorates PA-induced IR by enhancing autophagy.

SESN2, a member of the sestrin family, regulates metabolism to maintain redox homeostasis within the cell^35^. SESN2 exhibits antioxidative and autophagy-enhancing effects, protects cells from harmful stimuli, and plays a protective role in treating diseases associated with glucose and lipid metabolism^36^. Li et al.^37^ suggested that SESN2 effectively restores the impaired insulin signal transduction in skeletal muscles by enhancing autophagy. They found that autophagy activity significantly decreased following the administration of PA, whereas SESN2 overexpression reversed the inhibition of autophagy signaling caused by PA, which supports our findings. Our study showed that liraglutide effectively prevented IR in PA-exposed L6 myotubes by activating autophagy via SESN2. Silencing SESN2 by the corresponding siRNA inhibited the effects of liraglutide on impaired insulin signaling and autophagy. The results suggested that liraglutide decreased PA-induced L6 myotube IR through the regulation of SESN2; this is in accordance with a previous study^14^.

This study had some limitations. First, the interaction of liraglutide, SESN2, and autophagy in PA-induced IR in L6 myotubes was only examined, without considering other insulin signaling pathways. Secondly, in vivo studies are required to confirm the mechanism of action of liraglutide as a potential therapeutic target for the treatment of IR and T2DM. In skeletal muscles, targeting SESN2-related mechanisms that regulate glucose homeostasis might be an effective strategy.

## Conclusions

**Figure 7:**
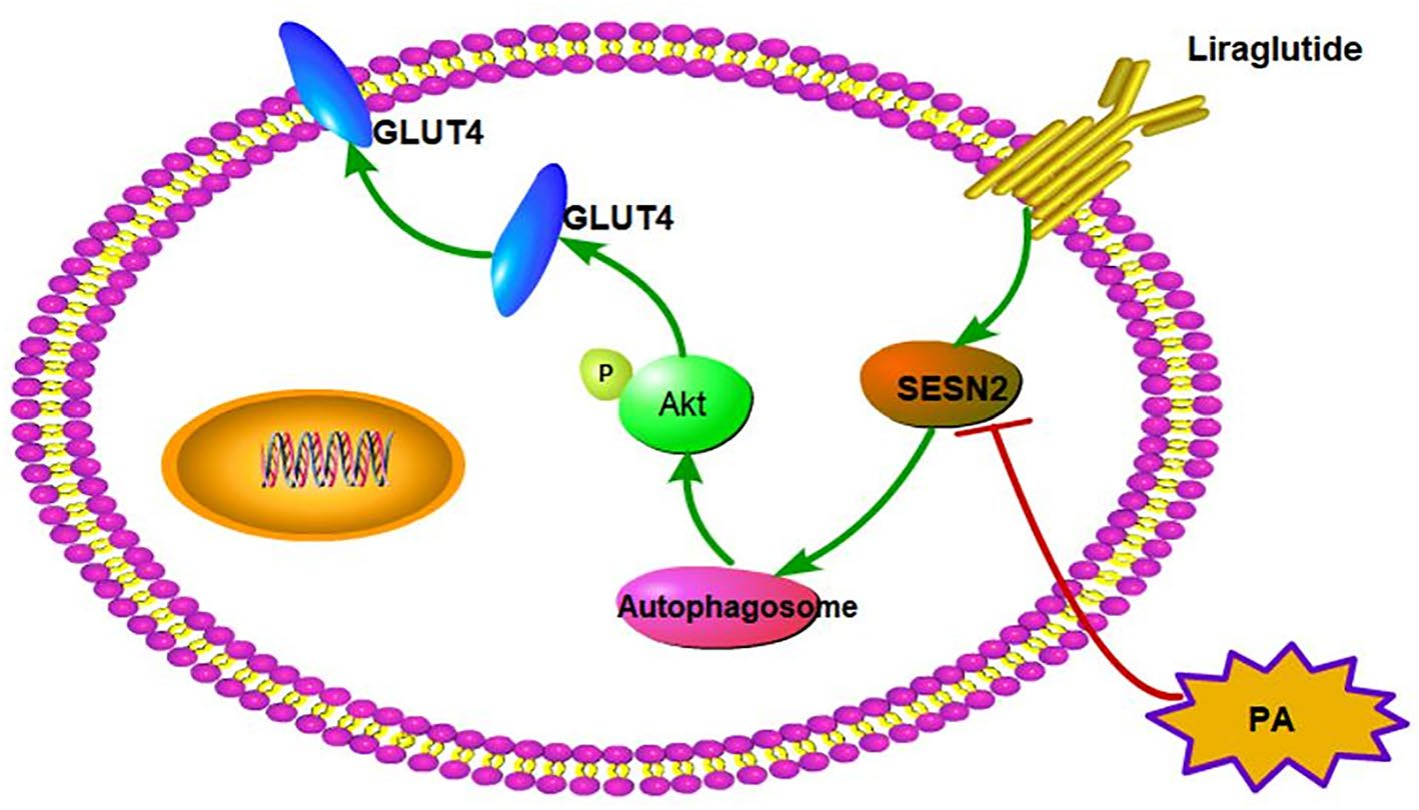
Schematic diagram of the mechanism of liralutide in reducing IR in skeletal muscle. PA suppresses SESN2 expression, leading to downregulation of autophagy which further impaired phosphorylation of Akt and expression of GLUT4. As a result, causing L6 cells IR. Liraglutide improved PA-induced IR in L6 myotubes by increasing autophagy-mediated by SESN2.

In conclusion, according to our knowledge, it is the first study that demonstrates the protective effect of liraglutide on L6 myotubes against PA-induced IR by upregulating autophagy via SESN2 (Fig. 7). A new theoretical basis and experimental support for the application of liraglutide in the treatment of diabetes may be provided by this study.

## Supplementary Information

Supplementary Information.

## Data Availability

The datasets used and/or analysed during the current study available from the corresponding author on reasonable request.
