# Supplementary material for: GLP‑1 receptor agonist protects palmitate-induced insulin resistance in skeletal muscle cells by up-regulating sestrin2 to promote autophagy
Source: Sci Rep. 2023 Jun 9;13:9446. doi: 10.1038/s41598-023-36602-6 (PMC10256699; doi:10.1038/s41598-023-36602-6)
Supplement: Supplementary file 1 — Supplementary Information. [file 41598_2023_36602_MOESM1_ESM.pdf]

Supplementary Figures

Figure2

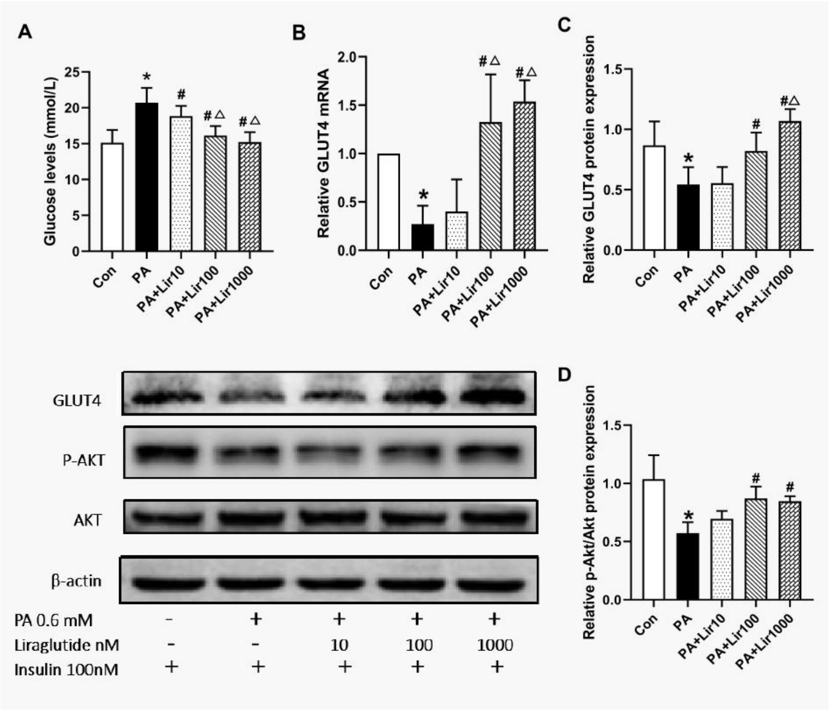

Figure S1

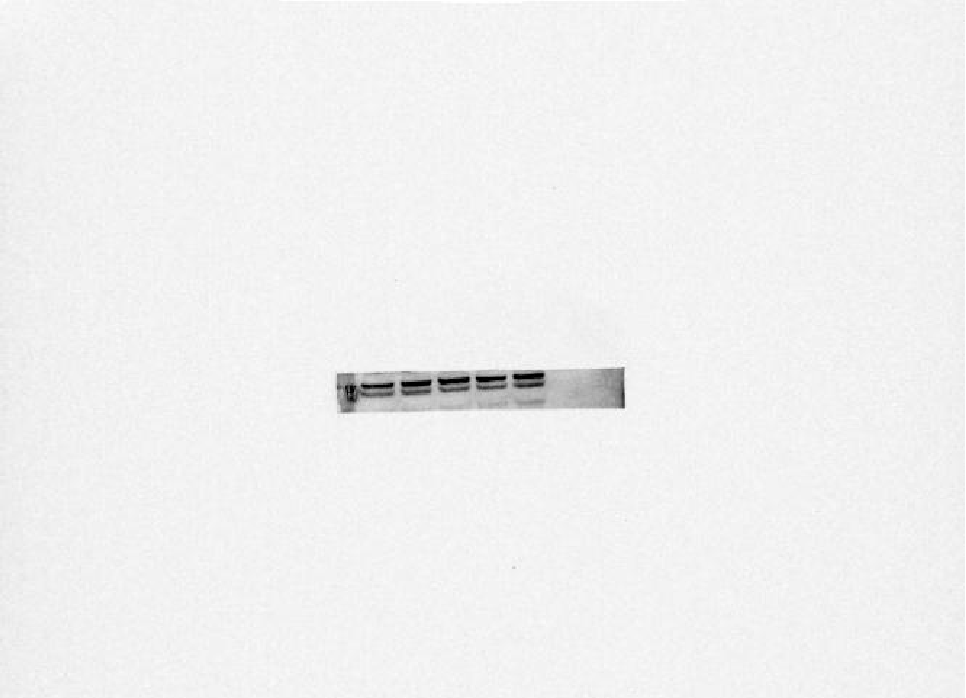

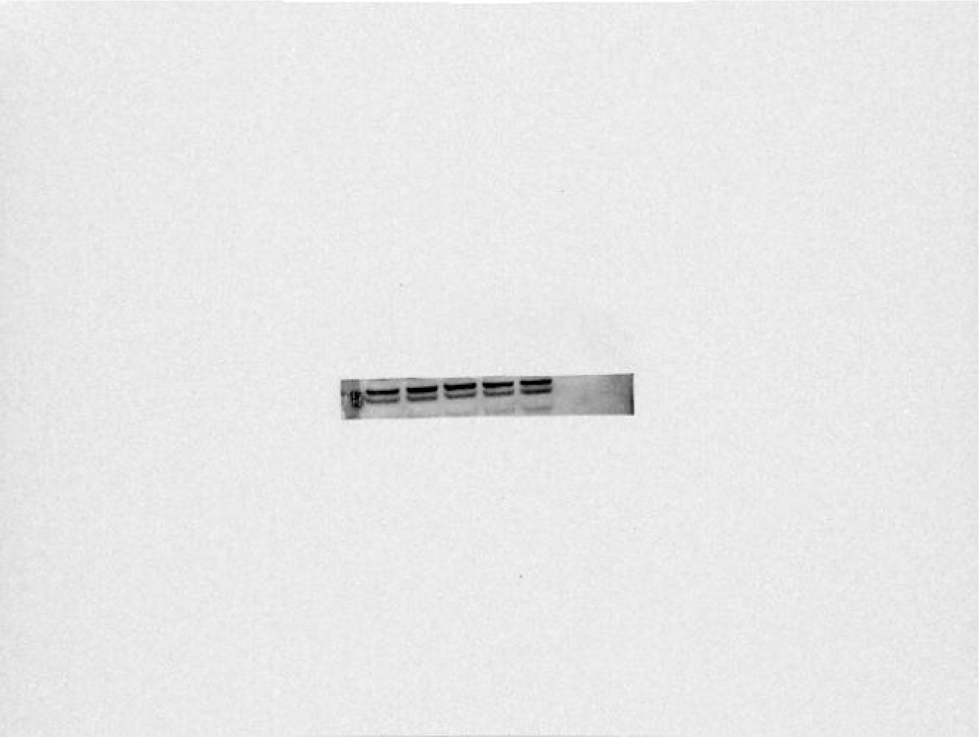

Figure S1 shows the gel and blotting of AKT in each group， from left to right are groups Con, PA, Lir10, Lir100 and Lir1000.　(gels/blots of AKT in Figure 2)

Figure S2

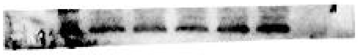

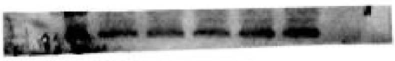

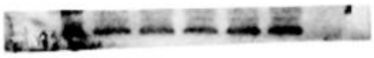

Figure S2 shows the gel and blotting of GLUT4 in each group, from left to right are groups Con, PA, Lir10, Lir100 and Lir1000. (gels/blots of GLUT4 in Figure 2)

Figure S3

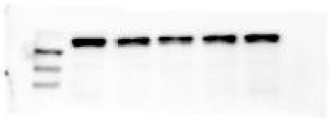

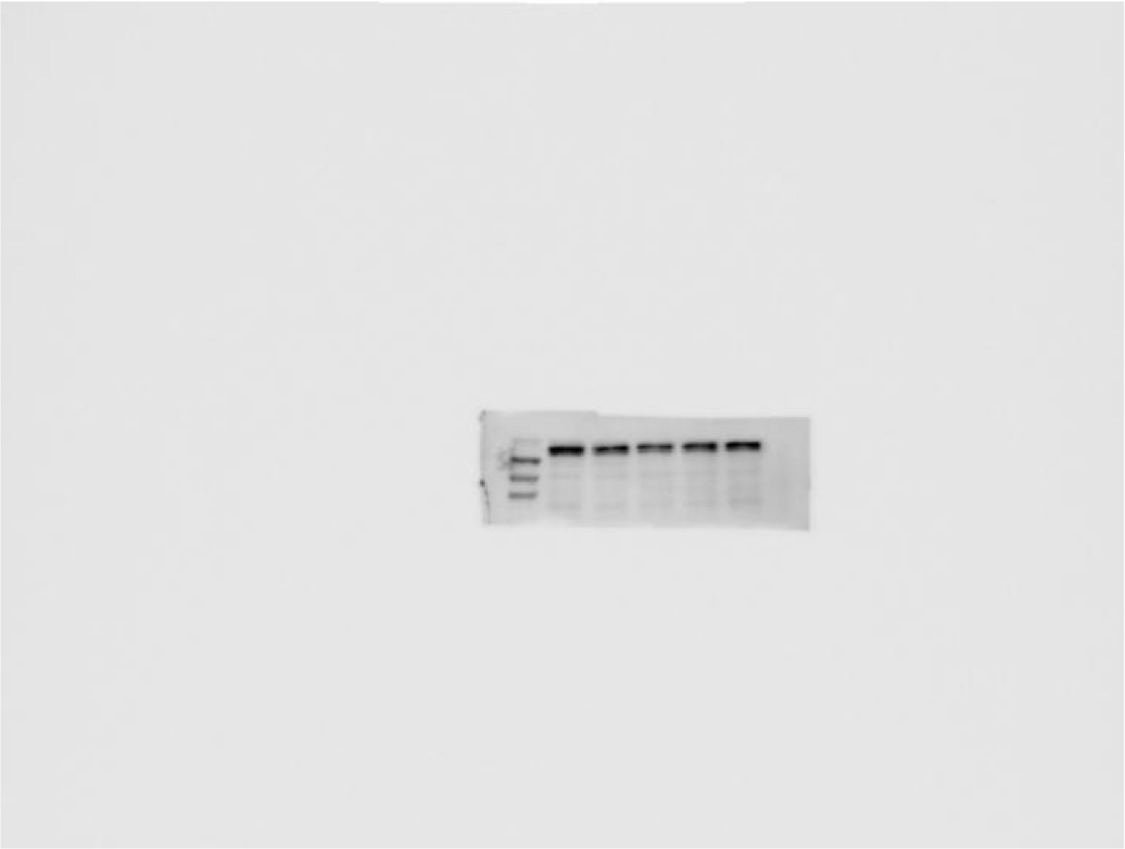

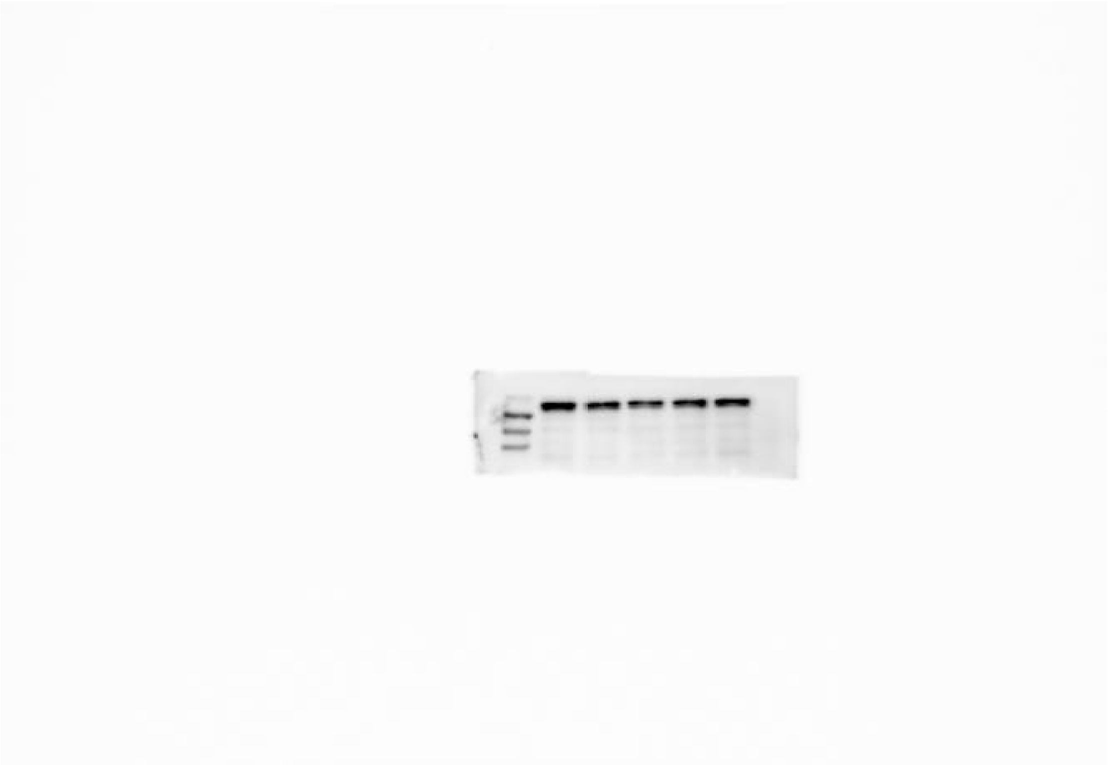

Figure S3 shows the gel and blotting of p-AKT in each group，from left to right are groups Con, PA, Lir10, Lir100 and Lir1000. (gels/blots of p-AKT in Figure 2)

Figure S4

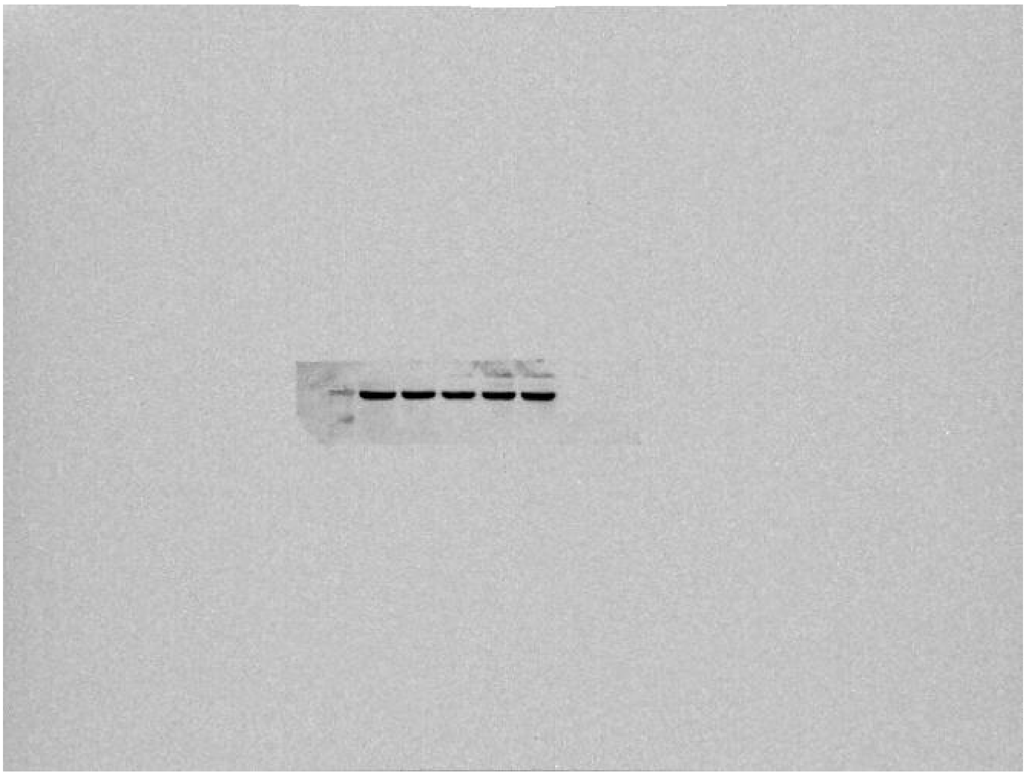

Figure S4 shows the gel and blotting of β-actin in each group, from left to right are groups Con, PA, Lir10, Lir100 and Lir1000. (gels/blots of β-actin in Figure 2)

Figure 3

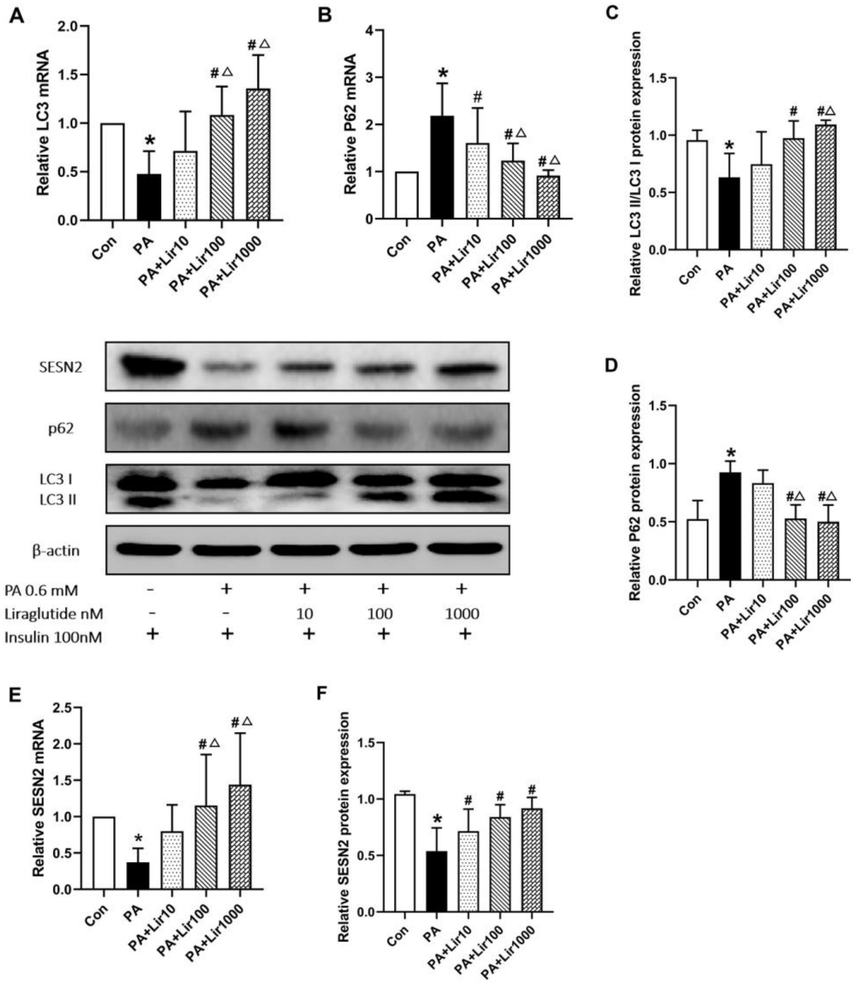

Figure S5

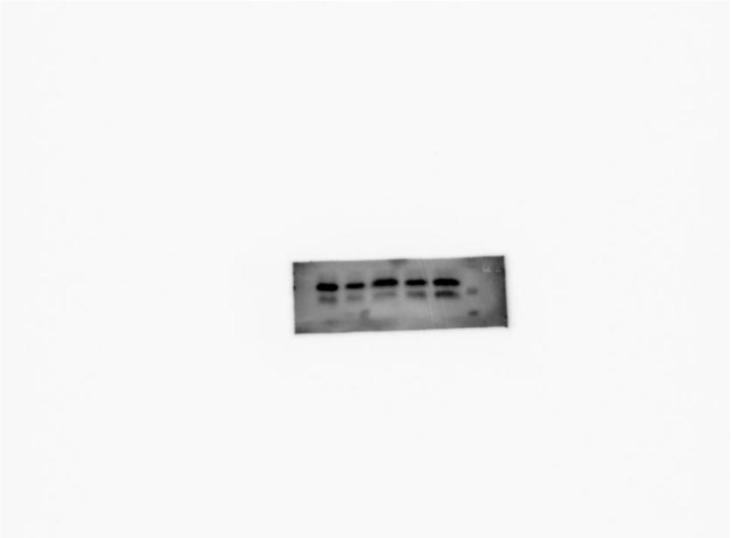

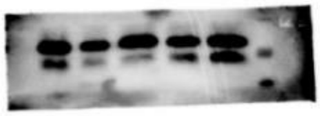

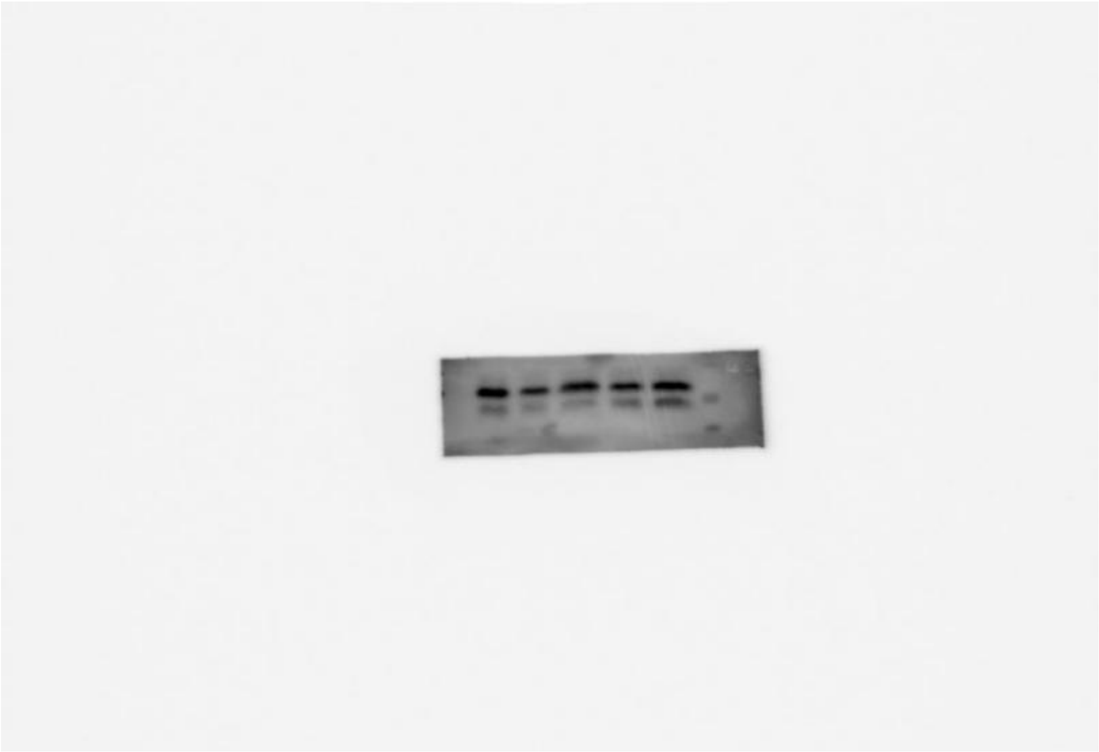

Figure S5 shows the gel and blotting of LC3 in each group， from left to right are groups Con, PA, Lir10, Lir100 and Lir1000. (gels/blots of LC3 in Figure 3)

Figure S6

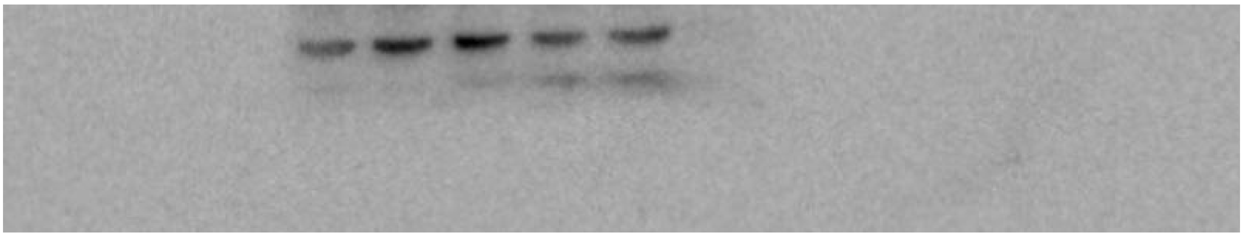

Figure S6 shows the gel and blotting of P62 in each group, from left to right are groups Con, PA, Lir10, Lir100 and Lir1000. (gels/blots of P62 in Figure 3)

Figure S7

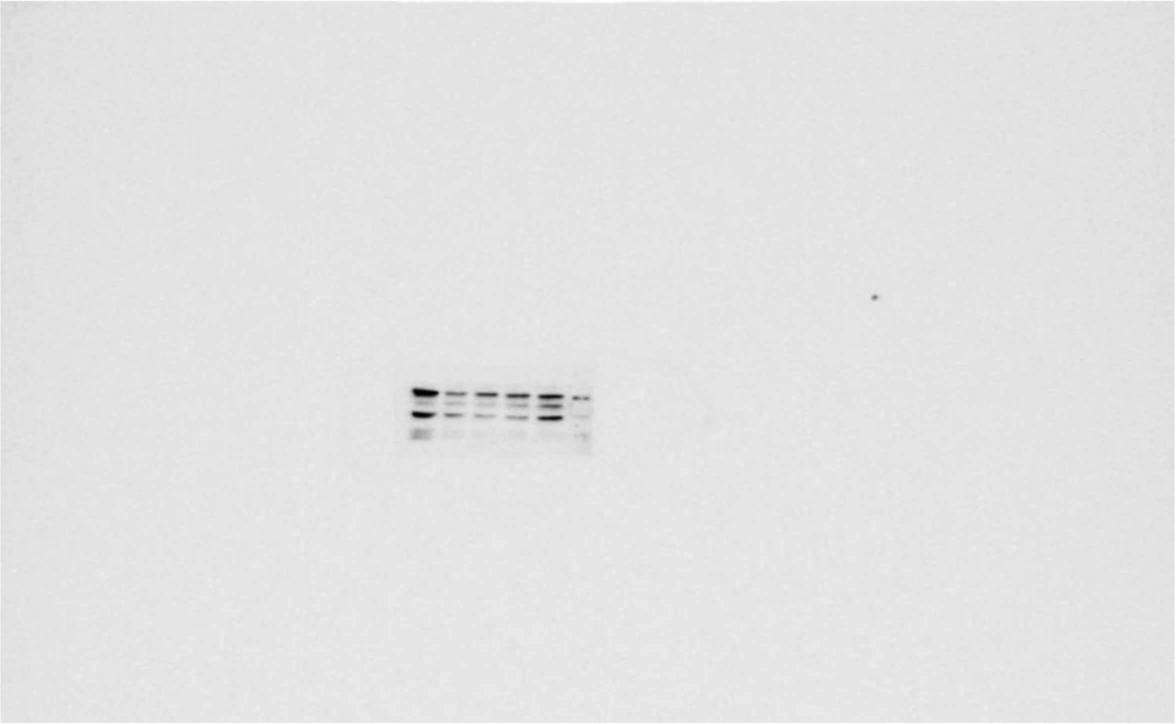

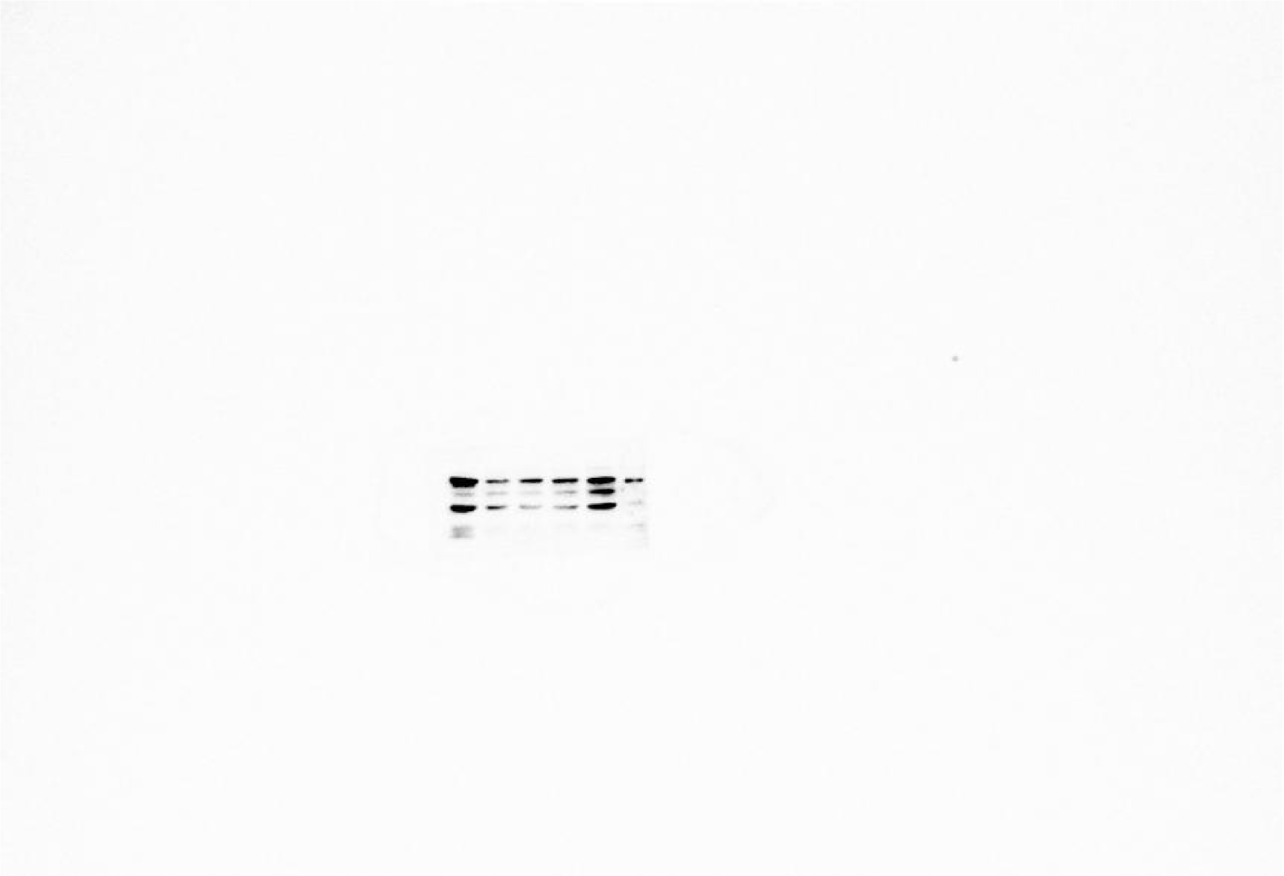

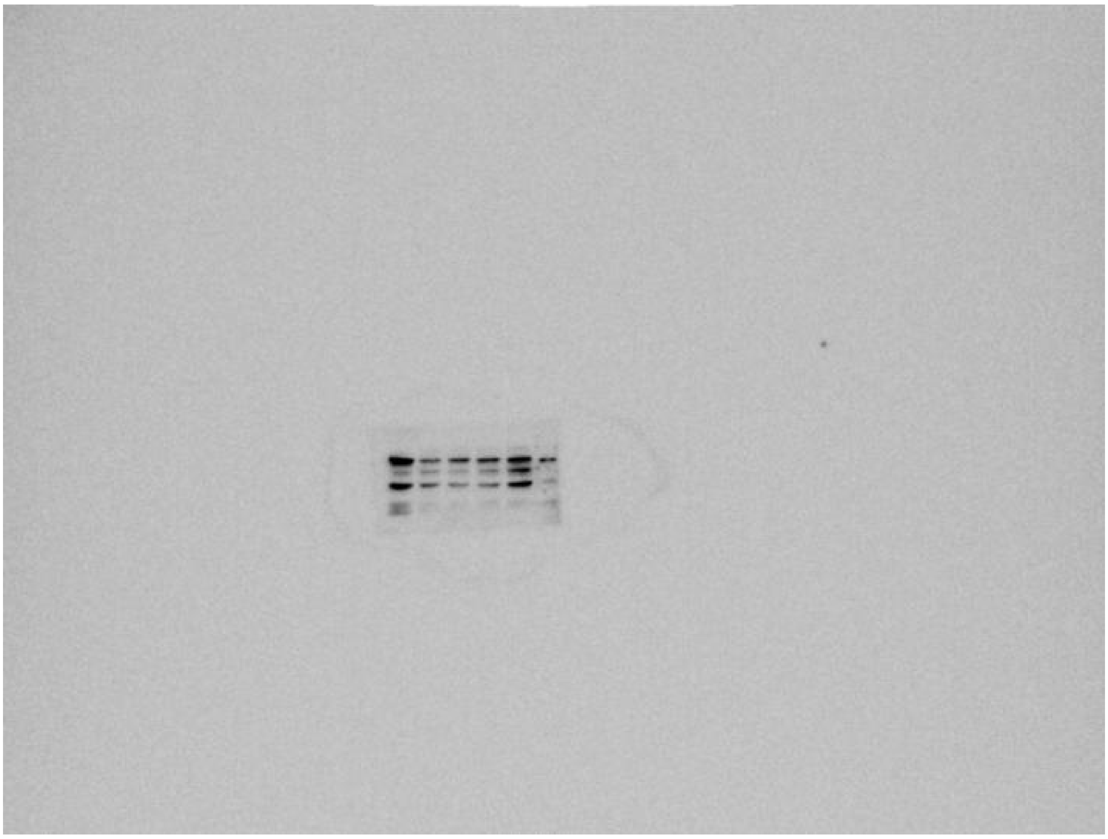

Figure S7 shows the gel and blotting of SESN2 in each group， from left to right are groups Con, PA, Lir10, Lir100 and Lir1000. (gels/blots of SESN2 in Figure 3)

Figure S8

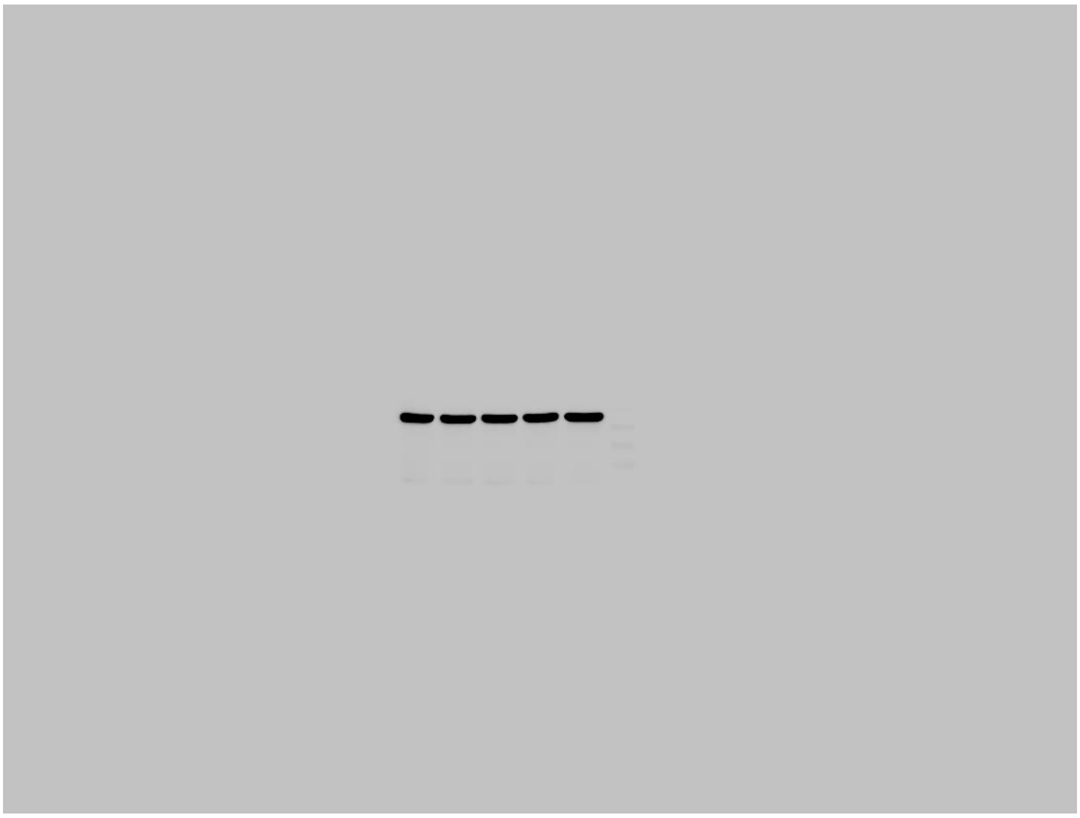

Figure S8 shows the gel and blotting of β-actin in each group, from left to right are groups Con, PA, Lir10, Lir100 and Lir1000. (gels/blots of β-actin in Figure 3)

Figure 4

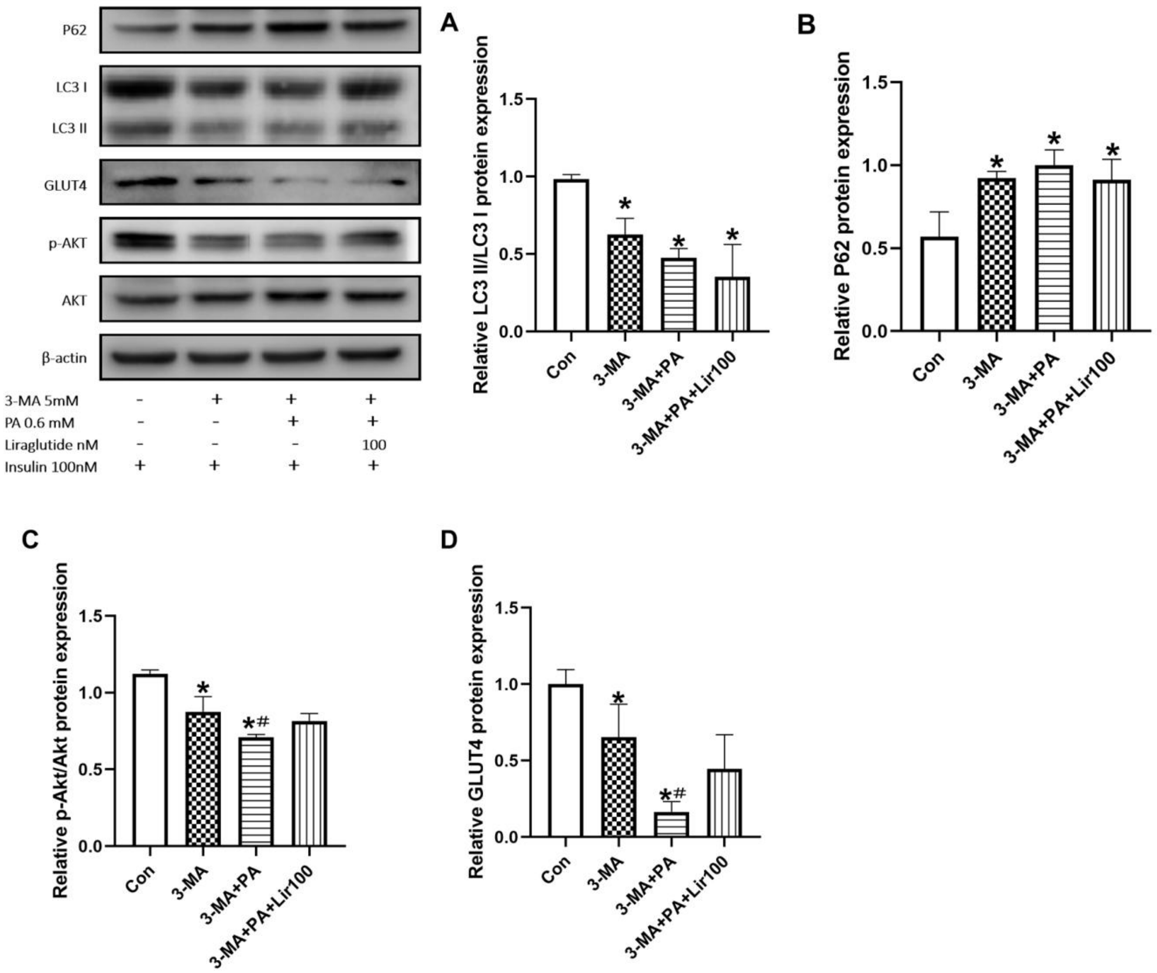

P62
LC3 I
LC3 II
GLUT4
p-AKT
AKT
β-actin
3-MA 5mM - + + +
PA 0.6 mM - - + +
Liraglutide nM - - - 100
Insulin 100nM + + + +
A
Relative LC3 II/LC3 I protein expression
1.5
1.0
0.5
0.0
Con
3-MA
3-MA+PA
3-MA+PA+Lir100
B
Relative P62 protein expression
1.5
1.0
0.5
0.0
Con
3-MA
3-MA+PA
3-MA+PA+Lir100
C
Relative p-Akt/Akt protein expression
1.5
1.0
0.5
0.0
Con
3-MA
3-MA+PA
3-MA+PA+Lir100
D
Relative GLUT4 protein expression
1.5
1.0
0.5
0.0
Con
3-MA
3-MA+PA
3-MA+PA+Lir100

Figure S9

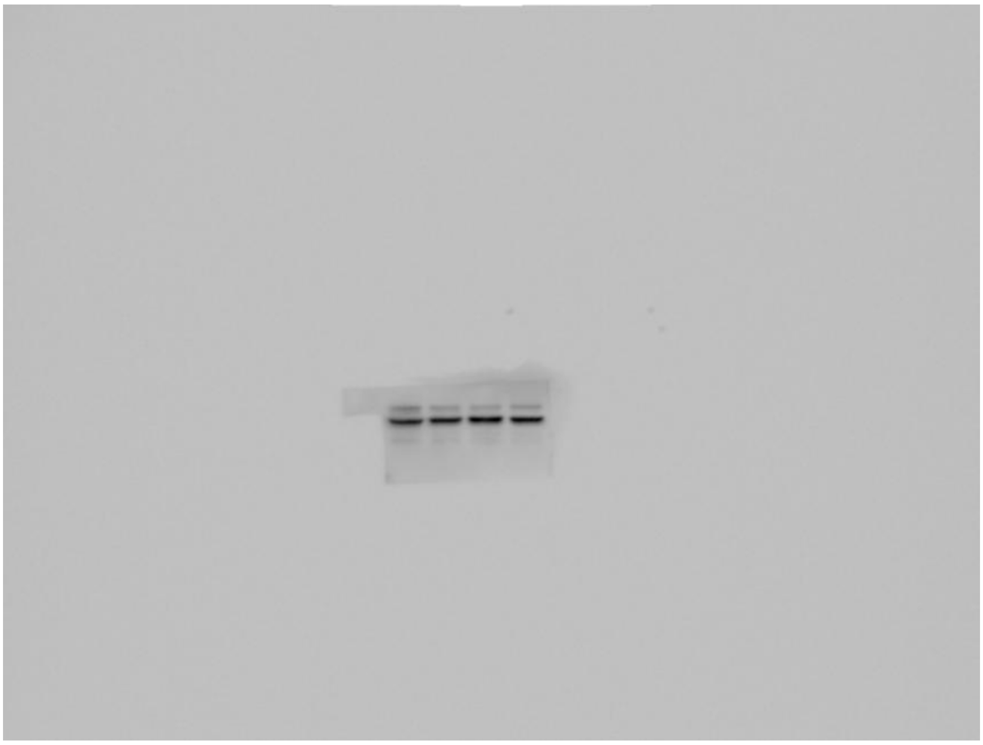

Figure S9 shows the gel and blotting of AKT in each group, from left to right are groups Con, 3-MA, 3-MA+PA and 3-MA+PA+Lir100. (gels/blots of AKT in Figure 4)

Figure S10

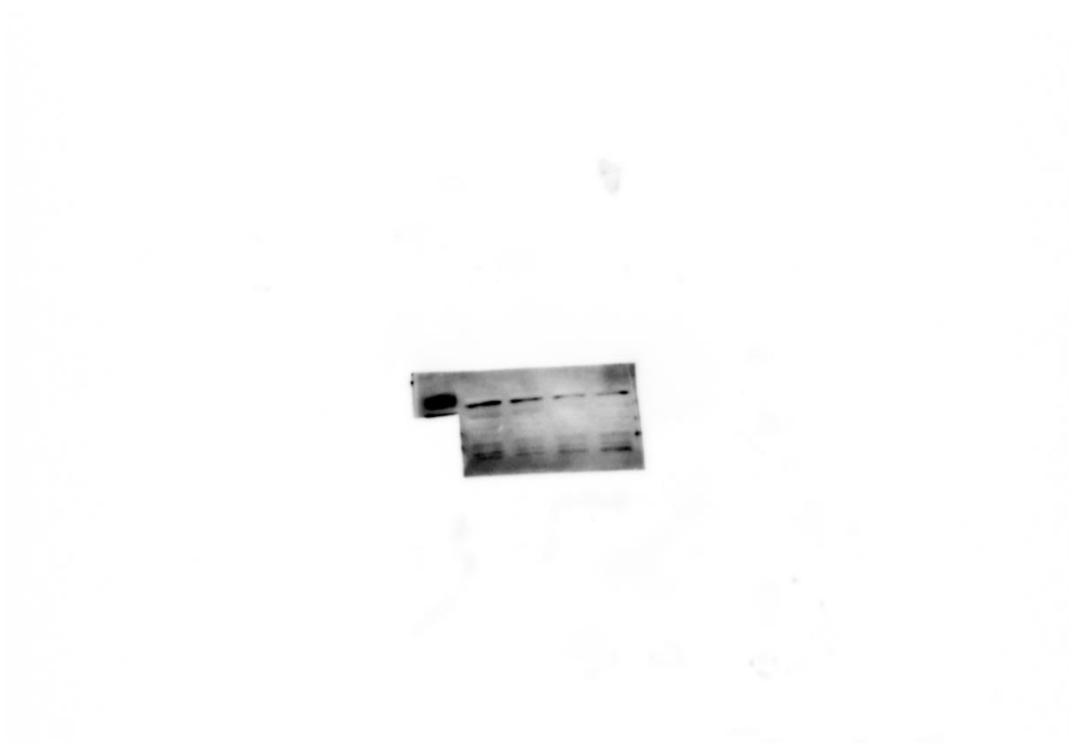

Figure S10 shows the gel and blotting of GLUT4 in each group， from left to right are groups Con, 3-MA, 3-MA+PA and 3-MA+PA+Lir100. (gels/blots of GLUT4 in Figure 4)

Figure S11

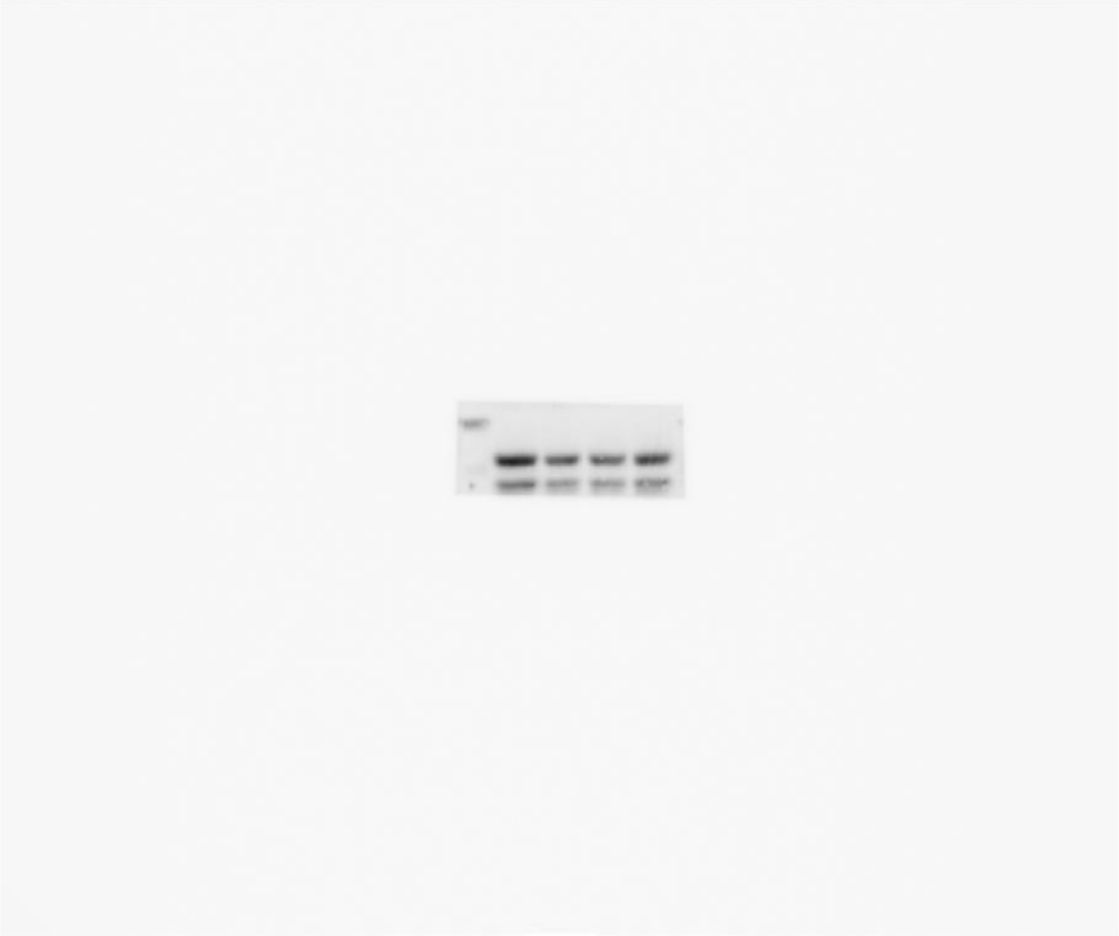

Figure S11 shows the gel and blotting of LC3 in each group， from left to right are groups Con, 3-MA, 3-MA+PA and 3-MA+PA+Lir100. (gels/blots of LC3 in Figure 4)

Figure S12

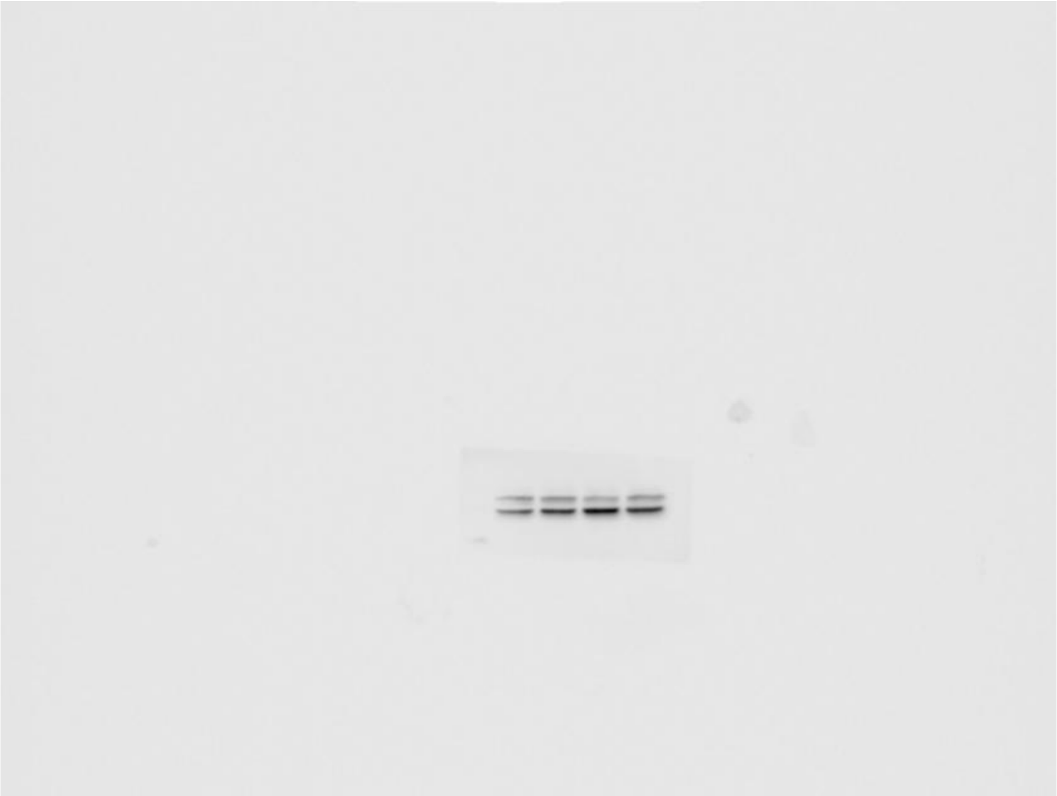

Figure S12 shows the gel and blotting of P62 in each group, from left to right are groups Con, 3-MA, 3-MA+PA and 3-MA+PA+Lir100. (gels/blots of P62 in Figure 4)

Figure S13

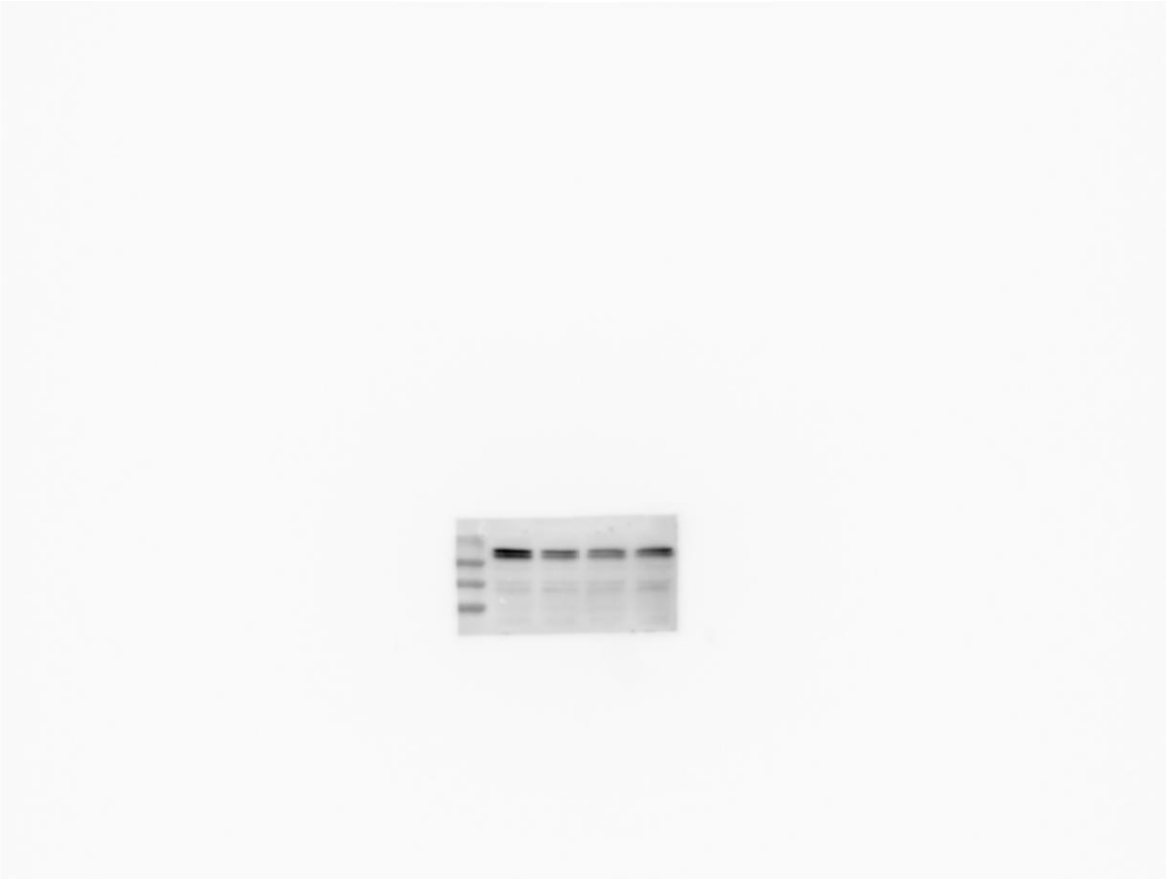

Figure S13 shows the gel and blotting of p-AKT in each group， from left to right are groups Con, 3-MA, 3-MA+PA and 3-MA+PA+Lir100. (gels/blots of p-AKT in Figure 4)

Figure S14

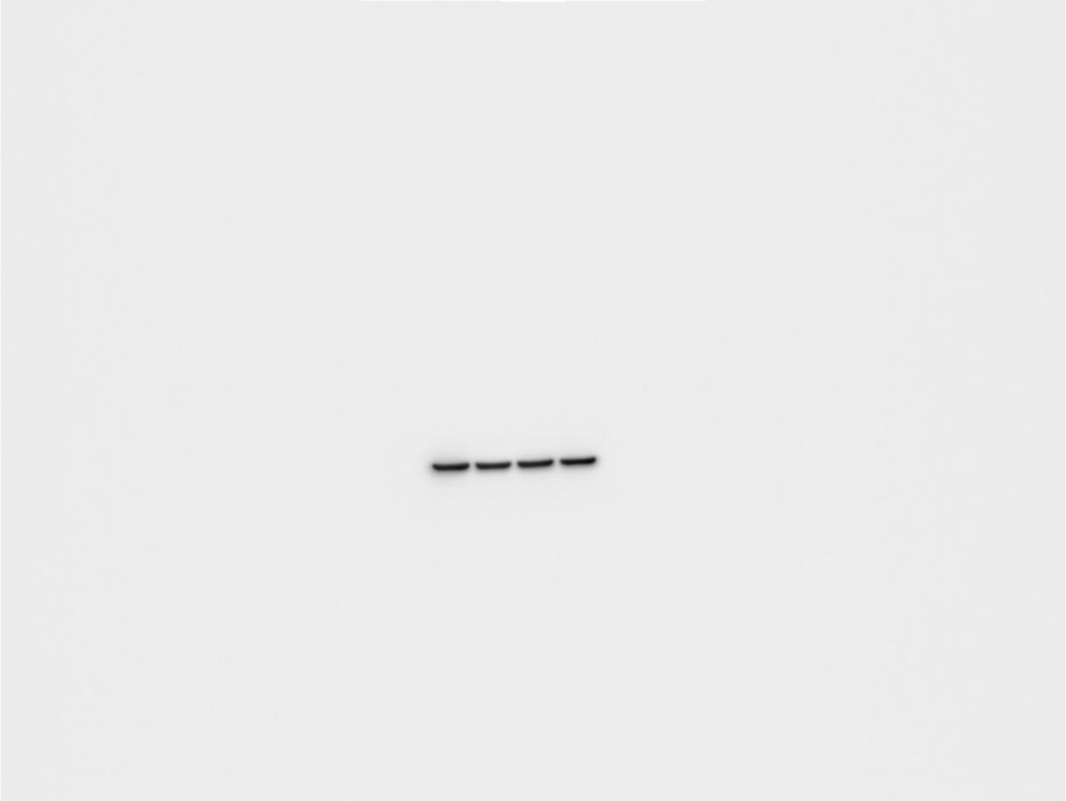

Figure S14 shows the gel and blotting of β-actin in each group， from left to right are groups Con, 3-MA, 3-MA+PA and 3-MA+PA+Lir100. (gels/blots of β-actin in Figure 4)

Figure 5

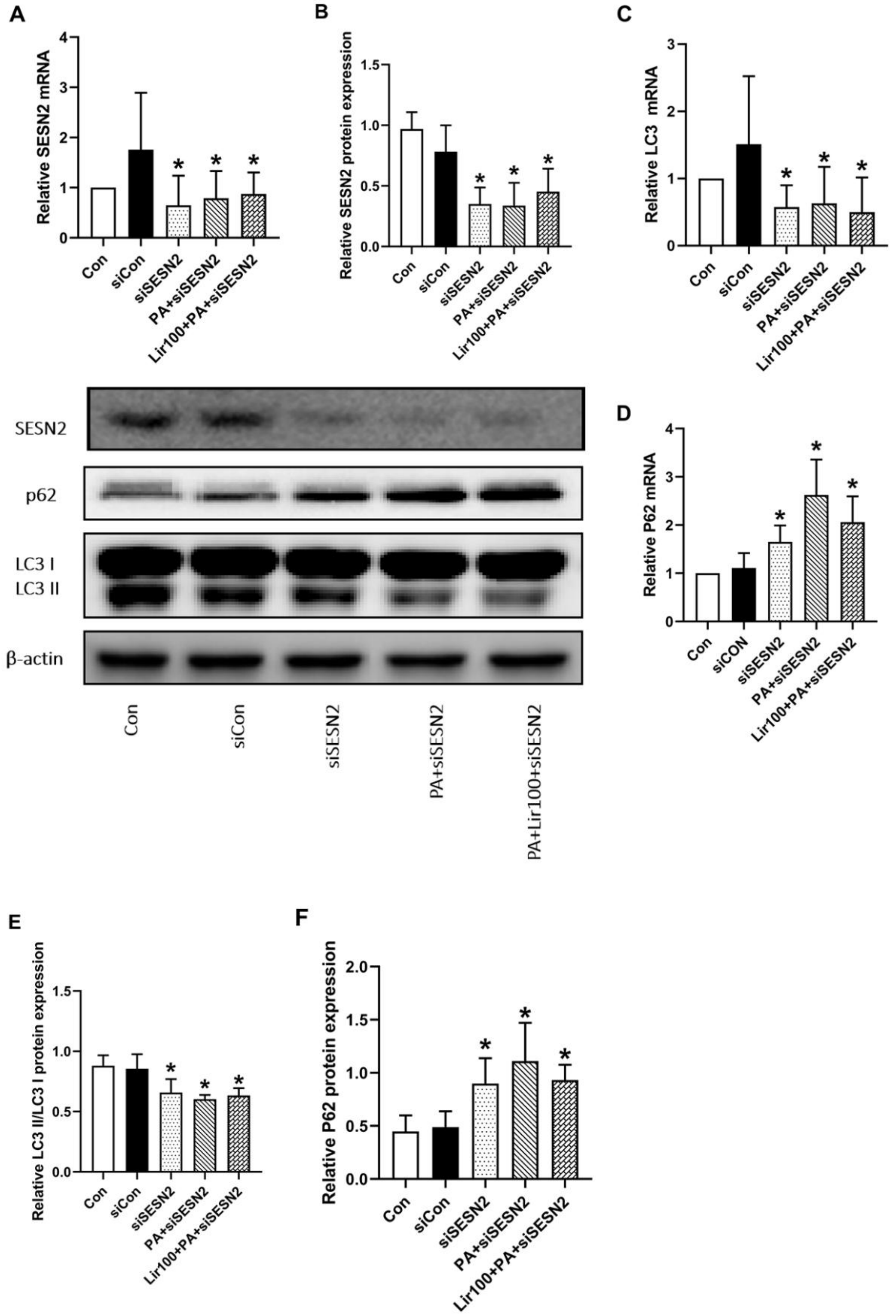
A
Relative SESN2 mRNA
4
3
2
1
0
*
*
*
Con
siCon
siSESN2
PA+siSESN2
Lir100+PA+siSESN2
B
Relative SESN2 protein expression
1.5
1.0
0.5
0.0
Con
siCon
siSESN2
PA+siSESN2
Lir100+PA+siSESN2
C
Relative LC3 mRNA
3
2
1
0
Con
siCon
siSESN2
PA+siSESN2
Lir100+PA+siSESN2
SESN2
p62
LC3 I
LC3 II
β-actin
Con
siCon
siSESN2
PA+siSESN2
PA+Lir100+siSESN2
D
Relative P62 mRNA
4
3
2
1
0
Con
siCON
siSESN2
PA+siSESN2
Lir100+PA+siSESN2
E
Relative LC3 II/LC3 I protein expression
1.5
1.0
0.5
0.0
Con
siCon
siSESN2
PA+siSESN2
Lir100+PA+siSESN2
F
Relative P62 protein expression
2.0
1.5
1.0
0.5
0.0
Con
siCon
siSESN2
PA+siSESN2
Lir100+PA+siSESN2

Figure S15

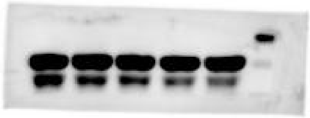

Figure S15 shows the gel and blotting of LC3 in each group, from left to right are groups Con, siCon, siSESN2, PA+siSESN2, and Lir100+PA+siSESN2. (gels/blots of LC3 in Figure 5)

Figure S16

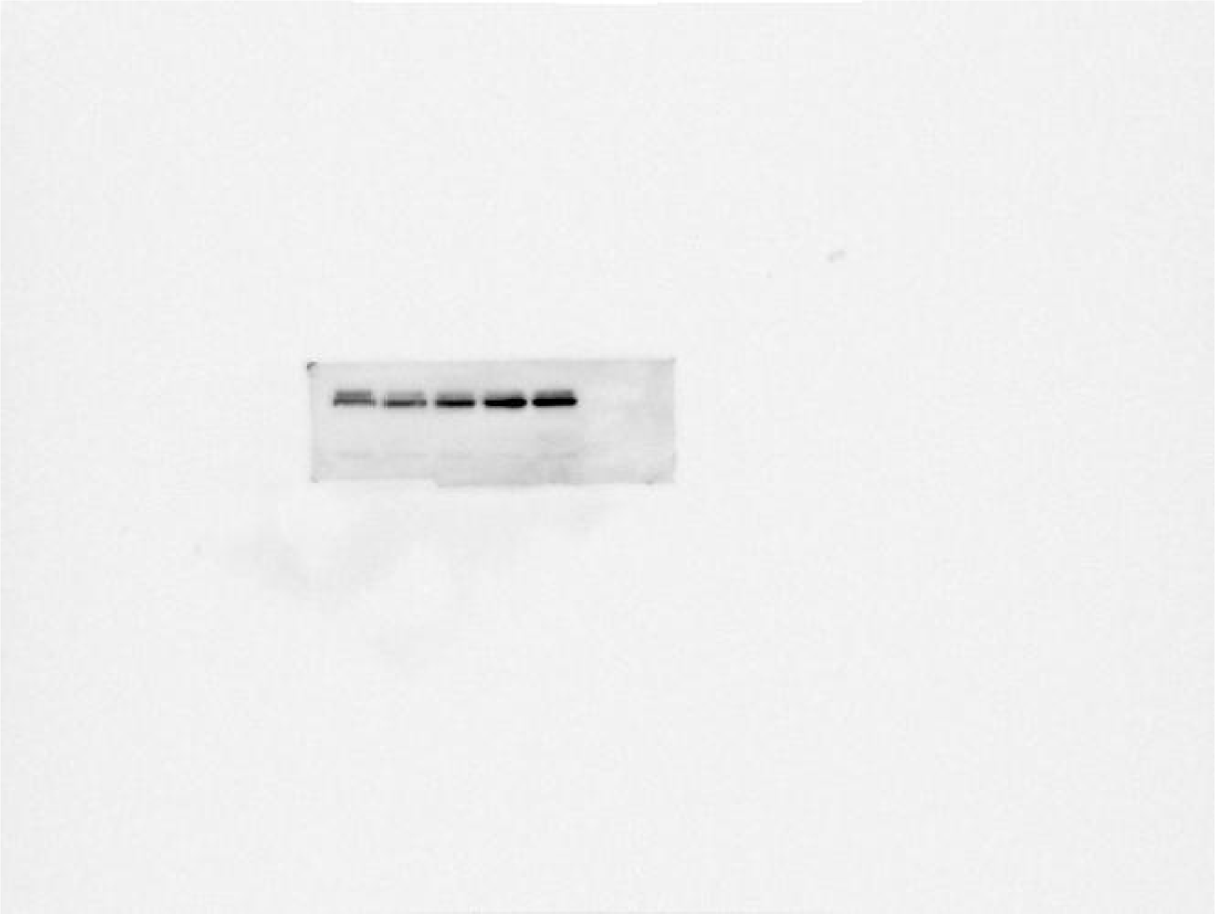

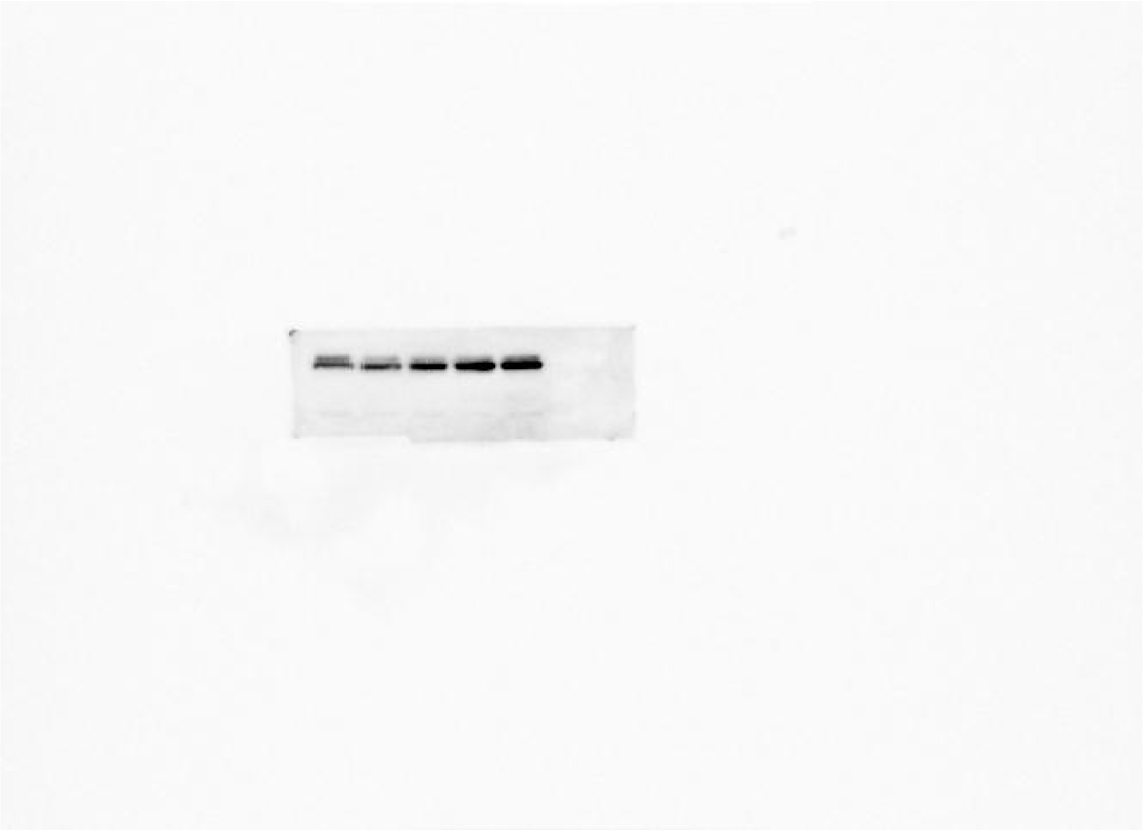

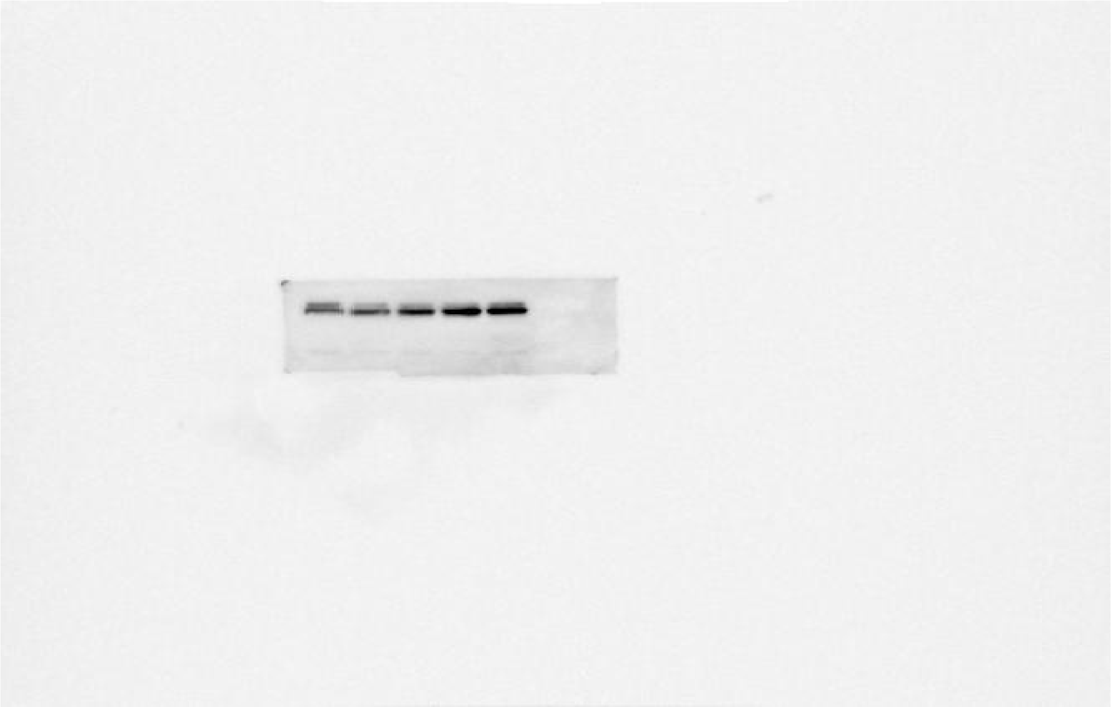

Figure S16 shows the gel and blotting of P62 in each group, from left to right are groups Con, siCon, siSESN2, PA+siSESN2, and Lir100+PA+siSESN2. (gels/blots of P62 in Figure 5)

Figure S17

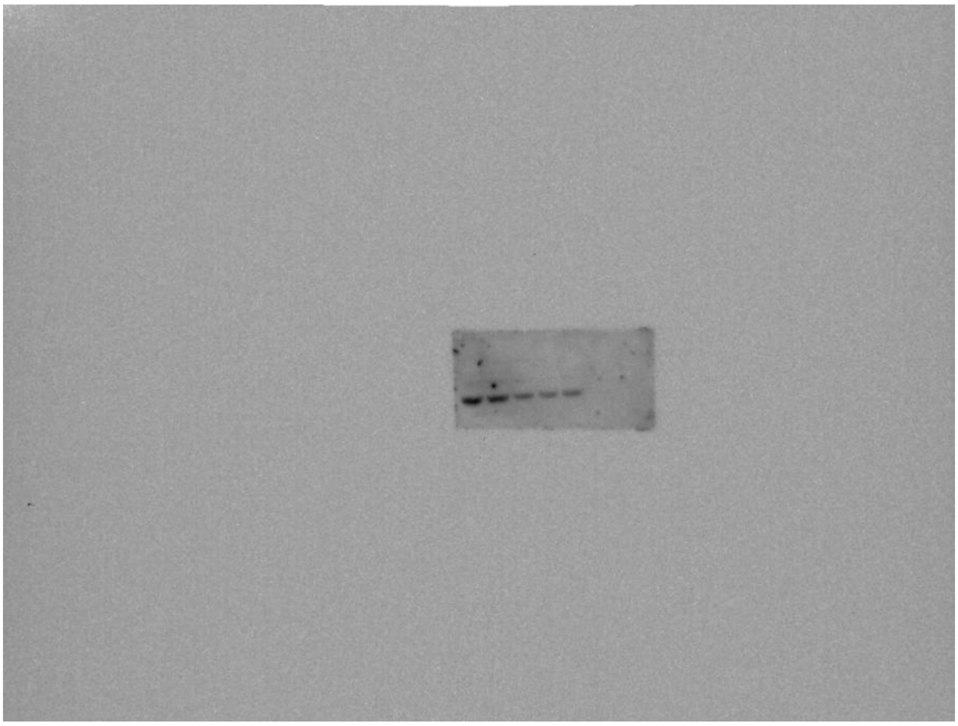

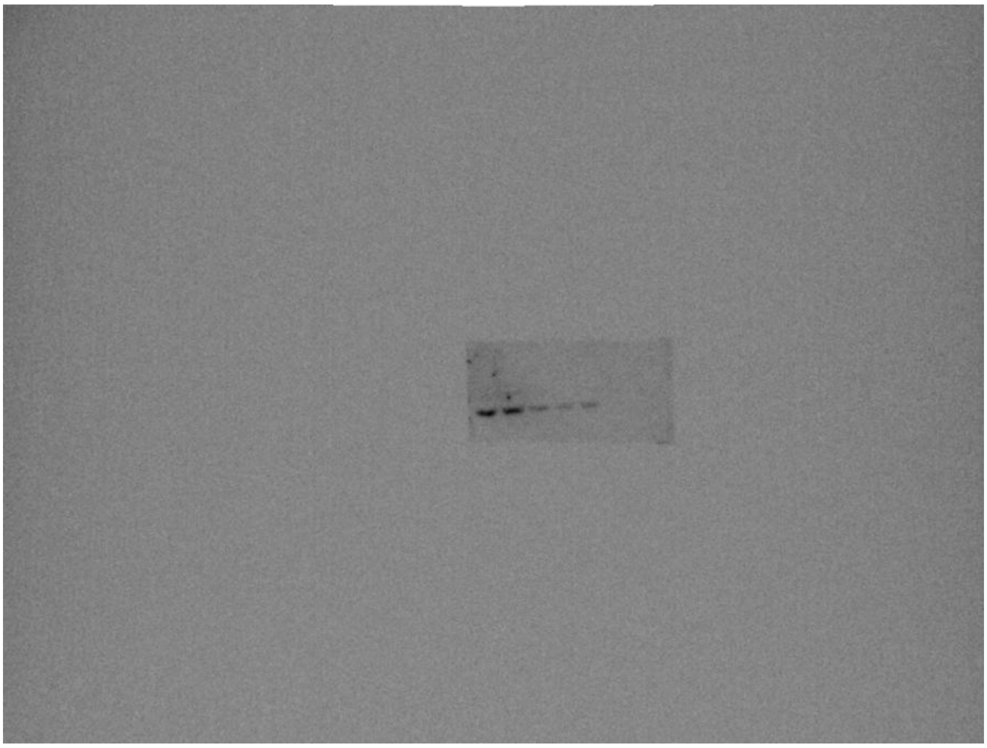

Figure S17 shows the gel and blotting of SESN2 in each group, from left to right are groups Con, siCon, siSESN2, PA+siSESN2, and Lir100+PA+siSESN2. (gels/blots of SESN2 in Figure 5)

Figure S18

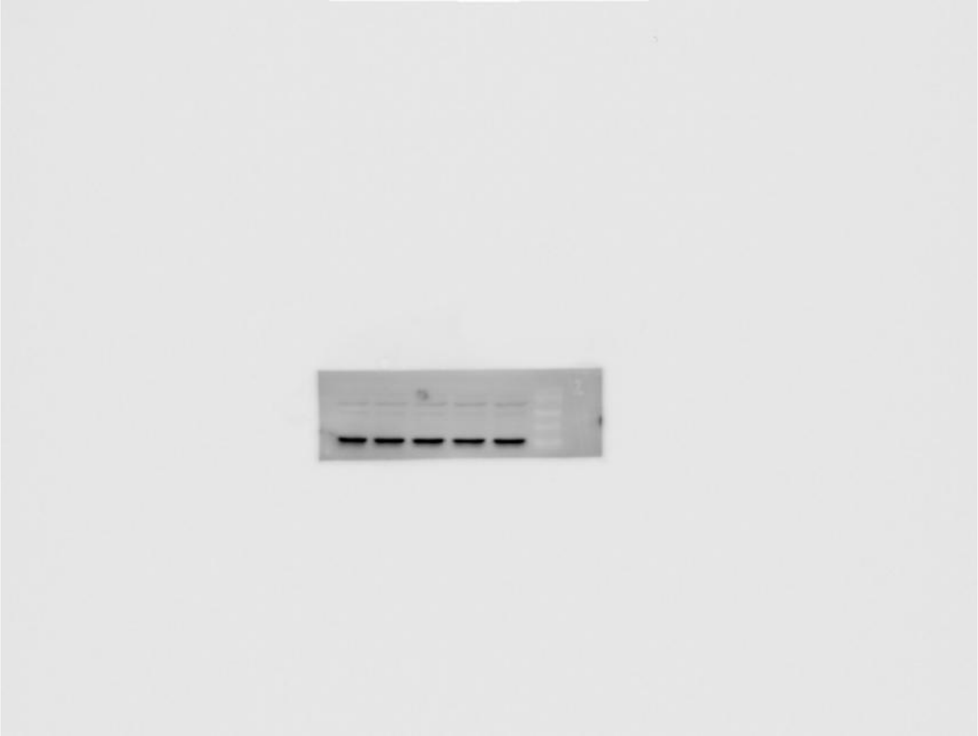

Figure S18 shows the gel and blotting of β-actin in each group, from left to right are groups Con, siCon, siSESN2, PA+siSESN2, and Lir100+PA+siSESN2. (gels/blots of β-actin in Figure 5)

Figure 6

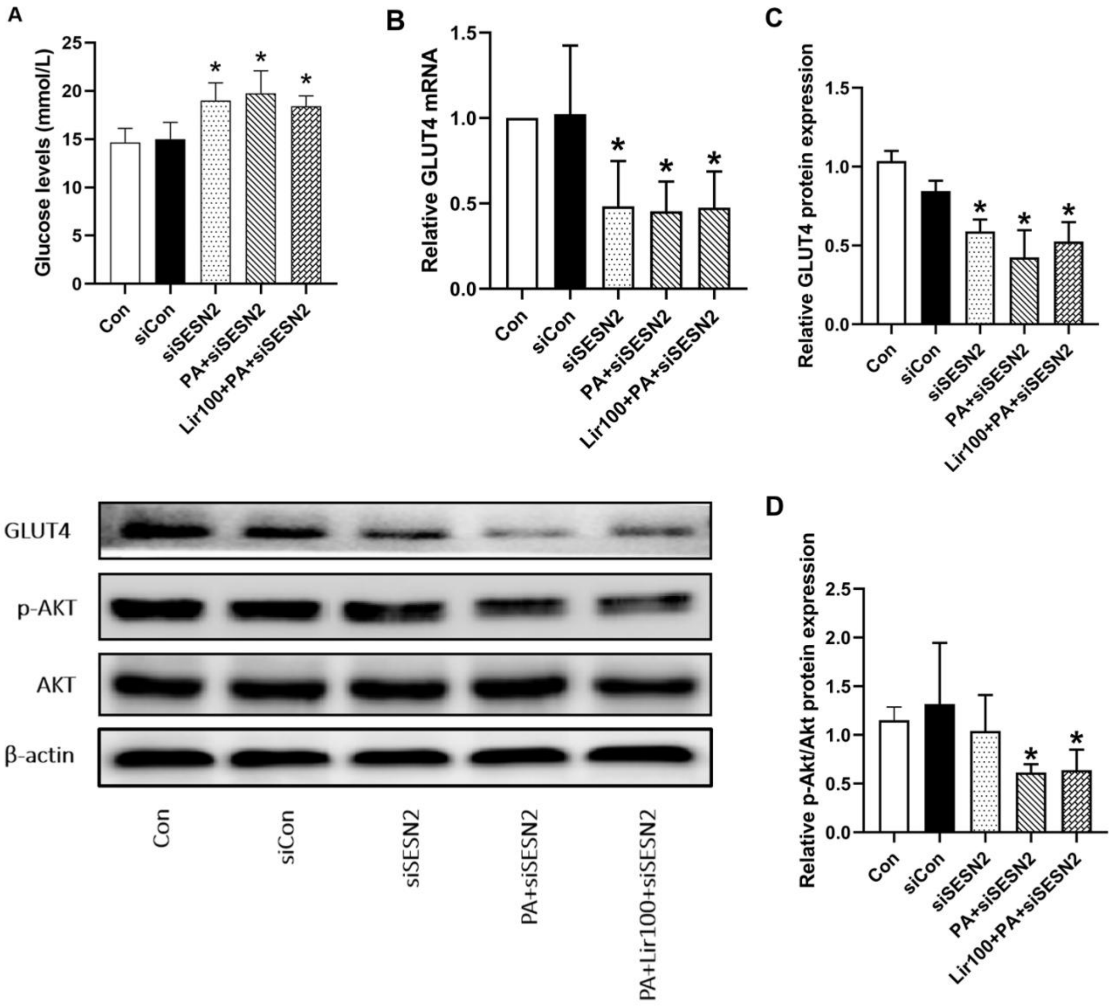
A
Glucose levels (mmol/L)
0
5
10
15
20
25
Con
siCon
siSESN2
PA+siSESN2
Lir100+PA+siSESN2
B
Relative GLUT4 mRNA
0.0
0.5
1.0
1.5
Con
siCon
siSESN2
PA+siSESN2
Lir100+PA+siSESN2
C
Relative GLUT4 protein expression
0.0
0.5
1.0
1.5
Con
siCon
siSESN2
PA+siSESN2
Lir100+PA+siSESN2
GLUT4
p-AKT
AKT
β-actin
Con
siCon
siSESN2
PA+siSESN2
PA+Lir100+siSESN2
D
Relative p-Akt/Akt protein expression
0.0
0.5
1.0
1.5
2.0
2.5
Con
siCon
siSESN2
PA+siSESN2
Lir100+PA+siSESN2

Figure S19

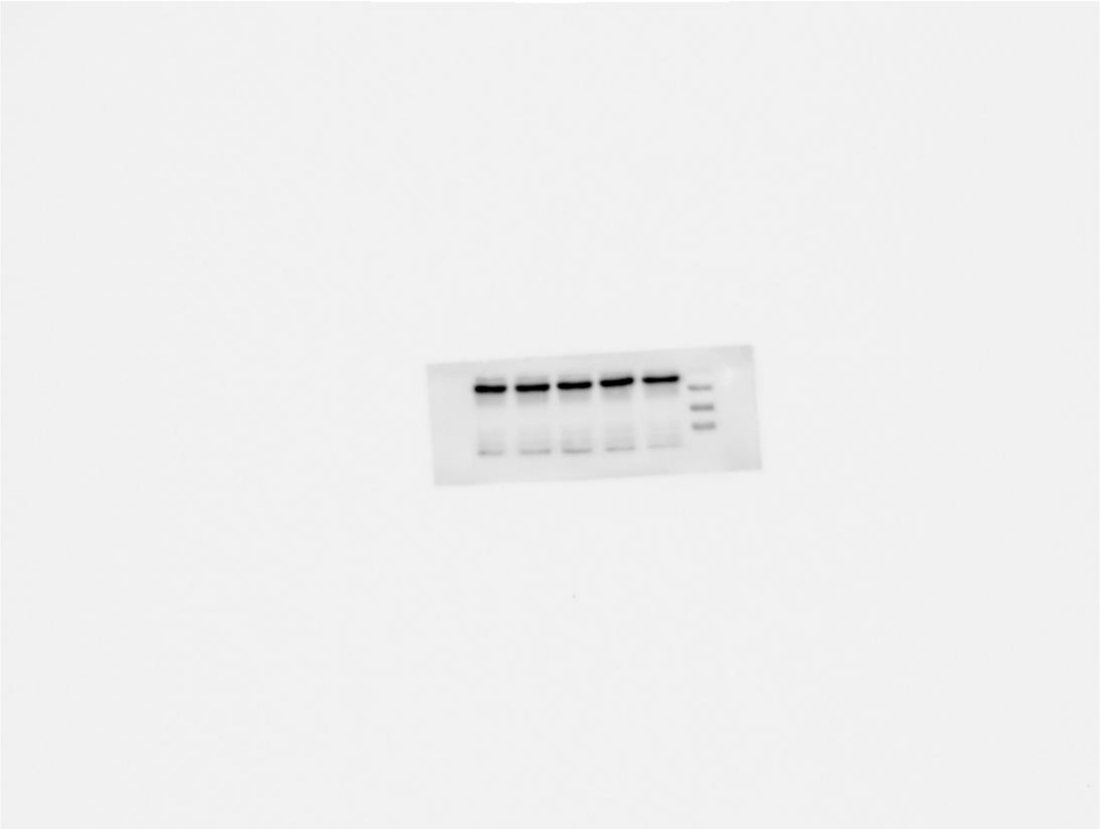

Figure S19 shows the gel and blotting of AKT in each group, from left to right are groups Con, siCon, siSESN2, PA+siSESN2, and Lir100+PA+siSESN2. (gels/blots of AKT in Figure 6)

Figure S20

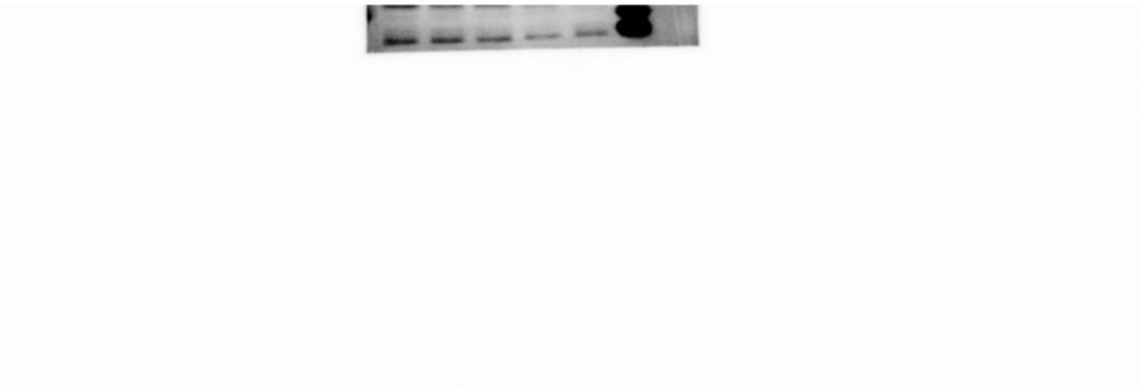

Figure S20 shows the gel and blotting of GLUT4 in each group， from left to right are groups Con, siCon, siSESN2, PA+siSESN2, and Lir100+PA+siSESN2. (gels/blots of GLUT4 in Figure 6)

Figure S21

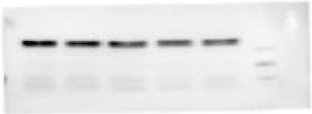

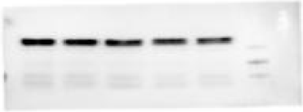

Figure S21 shows the gel and blotting of p-AKT in each group， from left to right are groups Con, siCon, siSESN2, PA+siSESN2, and Lir100+PA+siSESN2. (gels/blots of p-AKT in Figure 6)

Figure S22

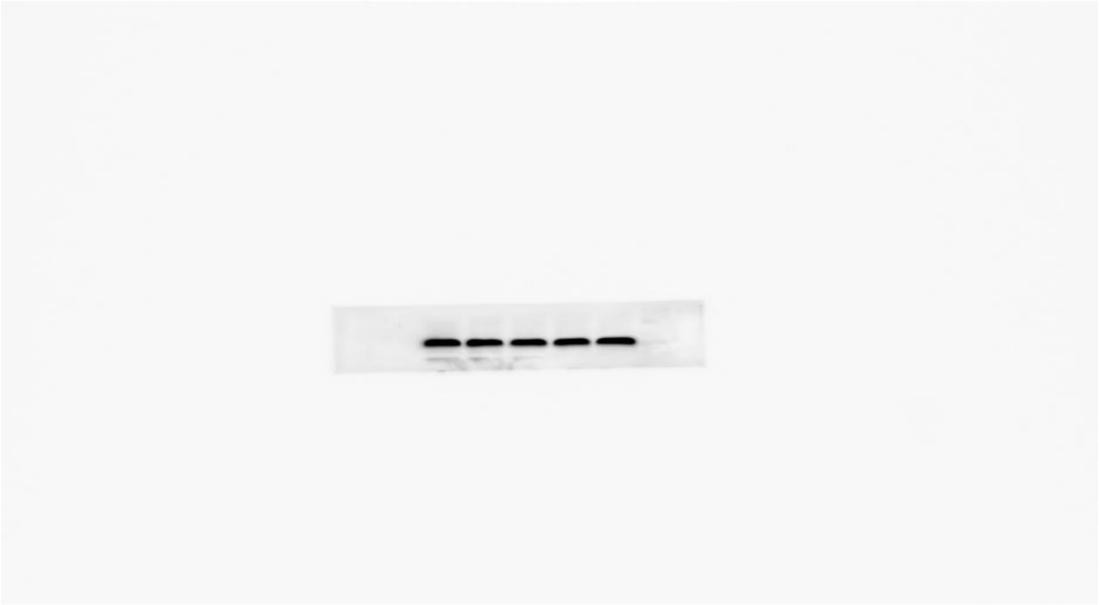

Figure S22 shows the gel and blotting of β-actin in each group, from left to right are groups Con, siCon, siSESN2, PA+siSESN2, and Lir100+PA+siSESN2. (gels/blots of β-actin in Figure 6)
